# Trajectories of macrophage ontogeny and reprogramming in cancer

**DOI:** 10.1016/j.isci.2025.112498

**Published:** 2025-04-22

**Authors:** Florent Duval, Joao Lourenco, Mehdi Hicham, Gaël Boivin, Alan Guichard, Celine Wyser-Rmili, Nadine Fournier, Nahal Mansouri, Michele De Palma

**Affiliations:** 1 Swiss Institute for Experimental Cancer Research (ISREC), School of Life Sciences, Swiss Federal Institute of Technology in Lausanne (EPFL), Lausanne, Switzerland; 2 Agora Cancer Research Center, Lausanne, Switzerland; 3 Swiss Cancer Center Léman (SCCL), Switzerland; 4 Translational Data Science (TDS) Facility, Swiss Institute of Bioinformatics (SIB), Lausanne, Switzerland; 5 Division of Pulmonary Medicine, Department of Medicine, Lausanne University Hospital (CHUV), University of Lausanne (UNIL), Lausanne, Switzerland

**Keywords:** Microenvironment, Immune response, Cancer, Transcriptomics

## Abstract

Tumor-associated macrophages (TAMs) often manifest immunosuppressive and tumor-promoting phenotypes contributing to immunotherapy resistance. *Dicer1* inactivation in TAMs (D^KO^) prompts their immunostimulatory activation, enabling effective immunotherapy in mouse cancer models. Single-cell RNA sequencing (scRNA-seq) analysis revealed interferon-γ (IFNγ)-dependent immunostimulatory programming of the tumor microenvironment in D^KO^ mice. In tumors of wild-type mice and patients with cancer, dynamic inferences on macrophage ontogeny by pseudotime analysis identified trajectories associated with monocyte-to-macrophage differentiation, progression into the cell cycle, and transition from immunostimulatory (M1-like) to immunosuppressive and protumoral (M2-like) states. *Dicer1* inactivation interfered with this trajectory and stalled TAMs at an intermediate state, impeding immunosuppressive and M2-like TAM development. This reprogramming translated into enhanced response to antiangiogenic immunotherapy in an orthotopic lung cancer model. Cycling/M2-like macrophages are conserved in mouse and human cancers and are enriched in patients with poor response to immunotherapy, making them a more selective therapeutic target than the bulk of TAMs.

## Highlights

• TAMs transition from immune-stimulatory to suppressive and cycling states
• Immune-suppressive and cycling TAMs are conserved in mouse and human cancer
• *Dicer1* inactivation stalls the trajectory of TAM differentiation
• *Dicer1* inactivation in TAMs stimulates antitumor immunity through IFNγ

## Graphical abstract

**Figure:**
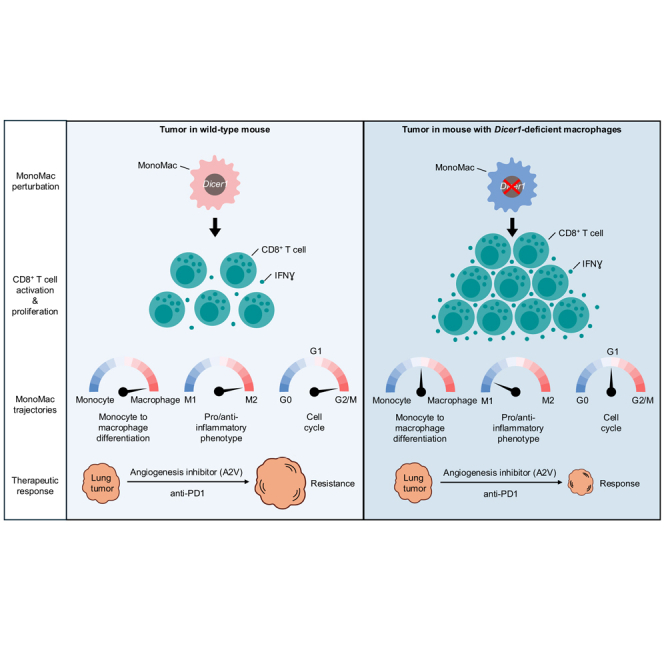

## Introduction

Macrophages residing in distinct tissue microenvironments display different phenotypes and functions.^1^ Such heterogeneity is dictated, in part, by the identity of the circulating monocytic or tissue-resident macrophage precursor from which the macrophages derive, and the microenvironmental factors to which they are locally exposed.^2^ When engaged, different receptors activate distinct intracellular molecular pathways that prompt a variety of differentiation trajectories and activation states in the macrophages. Mirroring Th1 and Th2 activation of lymphocytes, macrophages may be either classically (M1) or alternatively (M2) activated.^3^ Classically activated macrophages respond to interferon-γ (IFNγ) and bacterial toxins to instigate tissue inflammation, whereas alternatively activated macrophages respond to interleukin-4 (IL-4) and other Th2 cytokines to promote angiogenesis and wound healing. Classic and alternative activation represent extremes of a phenotypic continuum, and intermediate/mixed activation states largely predominate *in vivo*.^3^^,^^4^ In tumors, macrophages often acquire an alternatively activated, M2-like phenotype that is thought to enhance tumor growth and progression.^5^^,^^6^^,^^7^^,^^8^ Reprogramming tumor-associated macrophages (TAMs) toward an M1-like phenotype has been shown to limit tumor-associated immunosuppression, leading to improved anti-tumor immunity.^5^^,^^6^^,^^7^^,^^8^

Multiple cytokines expressed in the tumor microenvironment (TME), such as IL-4, IL-10, transforming growth factor β (TGF-β), tumor necrosis factor-α (TNF-α), CCL2, colony-stimulating factor 1 (CSF1), and vascular endothelial growth factor A (VEGFA), are known to regulate macrophage development, recruitment, differentiation, and/or M1/M2-like activation in cancer.^4^^,^^9^ Many of these cytokines also modulate microRNA (miRNA) expression and activity in different cell types, including monocytes and macrophages.^10^ Macrophages express measurable levels of several hundred miRNA species, some of which influence macrophage development, differentiation, and activation.^10^ We and others have previously reported that mice with an inactivating mutation in the miRNA-processing enzyme, *Dicer1*, specifically in myeloid cells exhibit delayed tumor growth in multiple cancer models.^11^^,^^12^ This effect was driven by the depletion of miRNAs (particularly Let-7 family miRNAs) in macrophages, which rewired prospective TAMs into an M1-like phenotype characterized by enhanced immunostimulatory functions.^11^ M1-like TAM programming then facilitated the recruitment and activation of IFNγ^+^CD8^+^ T cells that, in turn, enforced IFNγ-dependent M1-like TAM polarization to inhibit tumor growth. Here, we leveraged *Dicer1* inactivation in TAMs to study the ontogeny of M1- and M2-like macrophage states and their effects on the TME.

## Results

### Myeloid-specific *Dicer1* deletion reprograms macrophages, increases intra-tumoral CD8^+^ T cells, and delays tumor growth

**Figure 1:**
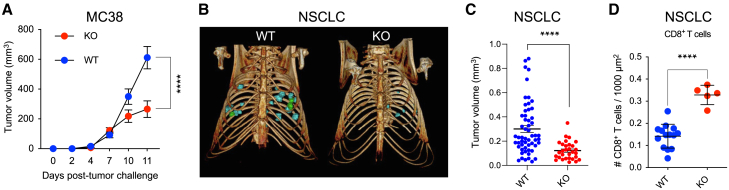
Myeloid-specific *Dicer1* deletion increases intra-tumoral CD8^+^ T cells and delays tumor growth (A) MC38 tumor volume (mean ± SEM) in D^WT^ (*n=6*) and D^KO^ (*n=5*) mice. Statistical analysis by two-way ANOVA with Sidak’s multiple comparison test. (B) Reconstructed computed tomography (CT) scan images of representative D^WT^ and D^KO^ mice imaged 28 days after inoculation of *Kras*^G12D^;*Tp53*^null^ NSCLC cells. (C) Individual tumor volumes (mean ± SEM) in the lungs of mice treated as indicated and imaged by micro-CT at day 28 post-tumor injection. Each dot represents a single tumor from D^WT^ (*n=4*) or D^KO^ (*n=5*) mice (*n* = 56 tumors in D^WT^; *n=30* in D^KO^ mice). Statistical analysis by unpaired Student’s t test. (D) Quantification of CD8^+^ T cells (mean ± SEM) by immunofluorescence staining of lung tumor sections of D^WT^ (*n=4*) and D^KO^ (*n=5*) mice (*n=14* tumors analyzed for D^WT^ and *n=5* for D^KO^). Note that D^KO^ mice had fewer lung tumors large enough for reliable analysis and quantification. Statistical analysis by unpaired Student’s t test. ∗∗∗∗*p* ≤ 0.0001.

We crossed *LysM*-Cre and *Dicer1*^lox/lox^ mice to obtain mice with biallelic inactivation of *Dicer1* specifically in myeloid-lineage cells (D^KO^ hereon).^11^ We then inoculated MC38 colon carcinoma cells subcutaneously in either *Dicer1* wild-type (D^WT^) or D^KO^ mice and observed delayed tumor growth in D^KO^ mice (Figure 1A), in agreement with previous studies.^11^ We also used an orthotopic model of *Kras*^G12D^;*Tp53*^null^ non-small cell lung cancer (NSCLC).^13^ Even in this aggressive tumor setting, D^KO^ mice exhibited significantly reduced incidence and growth of lung nodules (Figures 1B and 1C).

MC38 tumors in D^KO^ mice exhibited higher proportions of Ly6C^+^F4/80^-^ inflammatory monocytes but lower proportions of Ly6C^−^F4/80^+^ macrophages—particularly M2-like MRC1^+^ macrophages—compared to tumors in D^WT^ mice (Figures S1A and S1B). Moreover, both MC38 and NSCLC tumors had higher CD8^+^ T cell infiltration in D^KO^ mice (Figures 1D, S1A, and S1B). These results illustrate that myeloid-specific *Dicer1* inactivation inhibits tumor growth, (re)polarizes macrophages away from an M2-like phenotype, and enhances CD8^+^ T cell infiltration in the tumors.

### D^KO^ reprograms T cells and the TME through IFNγ

**Figure 2:**
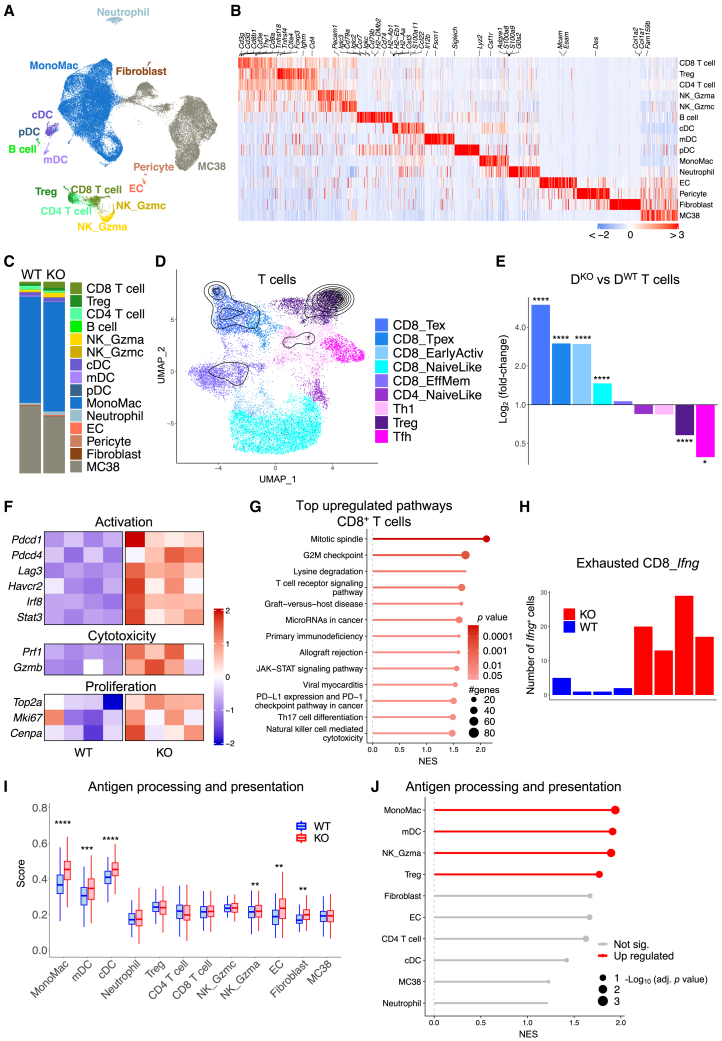
D^KO^ reprograms T cells and the TME through IFNγ (A) UMAP showing cell populations identified in MC38 tumors of D^WT^ and D^KO^ mice (pooled data; *n=4* per group) by scRNA-seq. (B) Expression of top enriched genes in the major cell populations of D^WT^ and D^KO^ mice (pooled data; *n=4* mice per group). Colors indicate *Z* scored gene expression. (C) Proportion of each cell population in D^WT^ and D^KO^ mice (*n=4*). (D) Projection of T cells onto the reference atlas of murine tumor-infiltrating T cell states from ProjecTILs (pooled data; *n=4* per group). Curved lines indicate cell density. (E) Analysis of the frequencies of T cell states in D^KO^ versus D^WT^ mice (log_2_ fold-change; *n=4*). For color codes, see (D). Statistical analysis by Pearson’s Chi-squared test. (F) Heatmap showing expression of the indicated genes in CD8^+^ T cells from individual D^WT^ and D^KO^ tumors (*n=4*). Color indicates scaled gene expression values (*Z* scores). (G) Lollipop plot showing the top upregulated (*p* < 0.05) Hallmark and KEGG pathways computed by gene set enrichment analysis (GSEA) in D^KO^ versus D^WT^ CD8^+^ T cells. The size of the circle corresponds to the number of genes in the pathway (#genes). NES: normalized enrichment score. (H) Number of exhausted CD8^+^ T cells expressing *Ifng* in individual tumors of D^WT^ and D^KO^ mice (*n=4*). (I) Hallmark gene set enrichment scores for the indicated biological process in the main cell populations of tumors of D^WT^ and D^KO^ mice (*n=4*). Boxes represent the interquartile range (IQR), horizontal lines represent the median, and whiskers represent the range within 1.5 times the IQR. Statistical analysis by Student’s t test. (J) Lollipop plot showing Hallmark GSEA of the indicated biological process across major cell populations. Genes were ranked according to log_2_ fold change between MC38 tumors of D^KO^ and D^WT^ mice (*n=4*). *p* values were adjusted for multiple testing according to the Benjamini-Hochberg method. ∗*p* ≤ 0.05, ∗∗*p* ≤ 0.01, ∗∗∗*p* ≤ 0.001, and ∗∗∗∗*p* ≤ 0.0001.

To investigate the effects of D^KO^-induced macrophage reprogramming on the TME, we conducted single-cell RNA sequencing (scRNA-seq) of MC38 tumors isolated from either D^WT^ or D^KO^ mice (*n* = 4 per group) at days 11–12 post-tumor challenge. We annotated different cell types using canonical-gene expression (Figures 2A, 2B, S2A and Table S1) and used an established signature to identify MC38 cancer cells.^14^ The monocyte/macrophage population (hereafter referred to as MonoMac), characterized by high *Lyz2* and *Csf1r* expression, comprised the majority of immune cells. In line with the flow cytometry data shown above, CD8^+^ T cells were enriched in D^KO^ tumors, whereas there was no significant difference in the abundance of MonoMac cells between the two groups (Figures 2C, S2B, and S2C).

To gain insights into T cell diversity in D^KO^ tumors, T cell clusters consisting of regulatory T cells (Treg), CD4^+^ and CD8^+^ T cells were resolved into sub-states by projecting the scRNA-seq data on a reference atlas of mutine T cells.^15^ Naive-like, precursor exhausted (Tpex), exhausted (Tex), and effector memory CD8^+^ T cells, as well as T_h_1 and Treg sub-states, were positively identified in both D^KO^ and D^WT^ tumors (Figure 2D and S3A). On the one hand, the majority of the activated CD8^+^ T cell sub-states were increased in D^KO^ tumors; on the other, Tregs—which represent the predominant T cell subset in MC38 tumors—were significantly decreased (Figure 2E). Differential gene expression and pathway enrichment analyses revealed upregulation in CD8^+^ T cells of genes associated with activation (such as *Pdcd1*, *Lag3*, and *Irf8*),^16^^,^^17^ cytotoxicity (*Prf1* and *Gzmb*),^18^ and proliferation (*Mki67*, *Top2a* and *Cenpa*),^19^ as well as pathways related to T cell activation and cell cycle progression, in D^KO^ compared to D^WT^ tumors (Figures 2F, 2G, and Table S1). Collectively, these results indicate that myeloid-specific *Dicer1* inactivation promotes intratumoral CD8^+^ T cell activation and proliferation.

IFNγ is a major effector of CD8^+^ T cell-mediated antitumor immunity.^20^ In an independent study, qPCR and ELISA revealed higher IFNγ abundance in MC38 tumor lysates and serum of D^KO^ mice compared to D^WT^ mice, respectively (Figures S3B and S3C). In the scRNA-seq study, *Ifng* was robustly expressed in both D^WT^ and D^KO^ tumors, while *Ifna* and *Ifnb* were not detected (Figures S3D and S3E). CD8^+^ T cells and granzyme A (*Gzma*)^hi^ natural killer (NK) cells were the main sources of *Ifng* in the tumors (Figure S4A); among CD8^+^ T cells, *Ifng* expression was higher in those with an exhausted phenotype. Notably, *Ifng-*expressing, exhausted CD8^+^ T cells were more abundant in D^KO^ than D^WT^ tumors (Figure 2H), potentially explaining the overall higher IFNγ production in D^KO^ tumors.

We next performed differential gene expression and pathway enrichment analyses between D^KO^ and D^WT^ tumors. Pathways regulated by interferons, such as antigen processing and presentation, IFNγ response, and T cell activation, were upregulated in D^KO^ tumors across multiple cell populations (Figures 2I, 2J, S4B, and S4C). These results suggest that myeloid-specific *Dicer1* deficiency reprogrammed the TME, at least in part, through exhausted CD8^+^ T cell-derived IFNγ.

### D^KO^ alters the abundance of inflammatory/M1-like and cycling/M2-like macrophages in tumors

**Figure 3:**
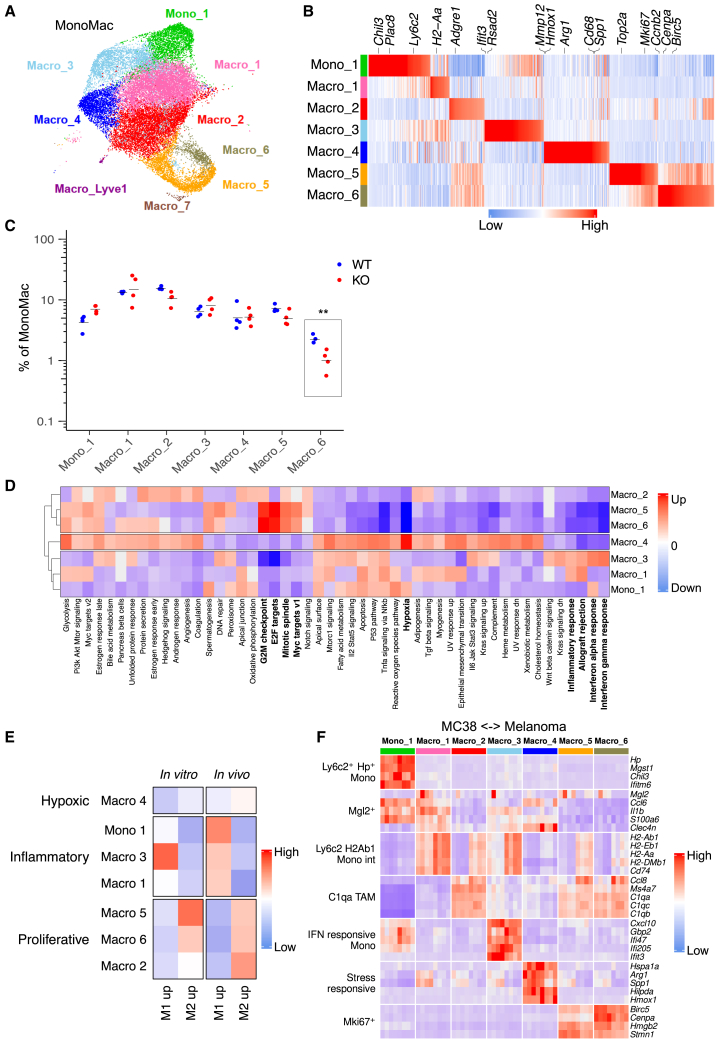
D^KO^ alters the abundance of inflammatory/M1-like and cycling/M2-like macrophages in tumors (A) UMAP showing clusters within the MonoMac population of MC38 tumors of D^WT^ and D^KO^ mice (pooled data; *n=4* per group). (B) Expression of top enriched genes in MonoMac subpopulations of MC38 tumors of D^WT^ and D^KO^ mice (pooled data; *n=4* per group). Colors indicate the Z-scored gene expression. (C) Abundance of MonoMac subpopulations in D^WT^ and D^KO^ tumors (*n=4*). Bars represent mean values. Statistical analysis by Student’s t test. (D) Heatmap showing NES of Hallmark gene-sets for each MonoMac subpopulation of MC38 tumors of D^KO^ and D^WT^ mice (pooled data; *n=4* per group). Enriched genes within MonoMac subpopulations were pre-ranked based on the average log2 fold-change. Colors represent NES: blue, negative NES values; white, NES = 0; red, positive NES values. Processes in bold font are related to cell proliferation and inflammation. (E) Signature scores for M1 and M2-like macrophage phenotypes derived from *in vitro*^21^ or *in vivo*^22^ datasets for each MonoMac subpopulation of MC38 tumors of D^WT^ and D^KO^ mice (pooled data; *n=4* per group). Colors indicate the scaled signatures scores. (F) Gene expression across MonoMac subpopulations in individual MC38 tumors of D^WT^ and D^KO^ mice (vertical lanes; *n=4* per group) using genes associated with monocyte and macrophage subsets identified in a mouse melanoma model (horizontal lanes; refer Mujal A.M. et al.^23^). Colors indicate the *Z* scored gene expression. ∗∗*p* ≤ 0.01.

We next studied the effects of *Dicer1* deletion on the MonoMac cells, which are the most abundant immune cells in MC38 tumors (see Figure 2C). We hypothesized that MonoMac comprised a heterogeneous cell population whose differential dynamics and plasticity in D^KO^ and D^WT^ tumors could influence tumor growth. We performed unsupervised clustering of the MonoMacs and identified 9 transcriptionally distinct clusters (or subpopulations) present in all samples regardless of *Dicer1* genotype (Figures 3A, 3B, S5A, and S5B; Table S1), which we annotated according to their gene signatures. Being exceedingly underrepresented, we excluded two clusters, Macro_7 (exhibiting a monocyte-derived dendritic cell signature) and Macro_Lyve1 (likely encompassing skin tissue-derived macrophages), from further analysis. We called the first cluster Mono_1, because it exhibited an immature monocyte signature characterized by high expression of *Ly6c2*, *Plac8*, and *Chil3*.^24^^,^^25^ Macro_1 and Macro_2 displayed less defined transcriptional signatures, suggesting an intermediate/transitional state between monocytes and macrophages. The Macro_3 cluster displayed enrichment of interferon-stimulated genes, including *Rsad2* and *Ifit3*,^26^ but shared similar gene expression patterns with Mono_1. Macro_4 expressed genes associated with hypoxia, such as *Spp1*, *Hmox1*, and *Mmp12*.^27^^,^^28^ Macro_5 and Macro_6, while transcriptionally related to Macro_2, exhibited upregulated cell cycle genes (*Mki67*, *Top2a*, and *Ccnb2*),^19^^,^^29^ indicative of a proliferative state. Notably, differential abundance analysis of the MonoMac subpopulations showed lower frequencies of proliferative Macro_6 in D^KO^ mice (Figure 3C).

We next performed differential gene expression and pathway enrichment analysis between each MonoMac subpopulation. Unsupervised clustering of the MonoMac according to Hallmark gene sets revealed two main groups based on their transcriptomic signatures: inflammatory monocytes-macrophages (Mono_1, Macro_1, and Macro_3) and cycling/proliferative macrophages (Macro_5, Macro_6 and, in part, Macro_2) (Figure 3D). Cycling macrophages displayed downregulated proinflammatory pathways, such as response to interferons, and upregulated cell cycle-associated biological processes. We then used two established signatures for M1 and M2 macrophage phenotypes derived from either *in vitro* or *in vivo* (tumor) settings.^21^^,^^22^ The inflammatory monocytes/macrophages exhibited a signature similar to M1-polarized macrophages, whereas cycling macrophages were more M2-like (Figures 3E, S5C, and S5D). These findings suggest the co-existence of diverse MonoMac subpopulations with potentially opposing functions in MC38 tumors.

Finally, we compared the MonoMac subpopulations observed in MC38 tumors with monocyte and macrophage subsets identified previously in a mouse melanoma model.^23^ Phenotypically overlapping monocyte/macrophage subsets were found in the two tumor models, with “Mki67^+^ macrophages” in melanoma corresponding to Macro_5 and Macro_6 in MC38 tumors, and “IFN responsive monocytes” in melanoma corresponding to Mono_1 and Macro_3 in MC38 tumors (Figure 3F). These results reveal partially conserved monocyte/macrophage subpopulations in two developmentally distinct mouse tumor models.

### Dynamics analysis links trajectories and M1/M2 phenotypes in MonoMacs

**Figure 4:**
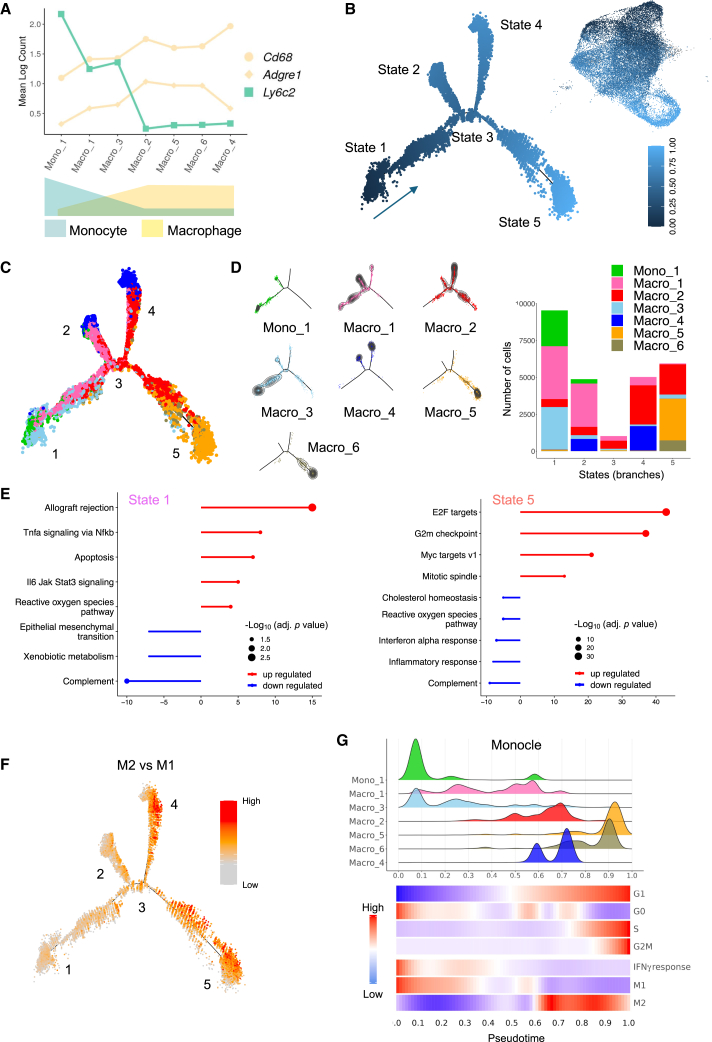
Dynamics analysis links trajectories and M1/M2 phenotypes in MonoMacs (A) Expression of selected marker genes indicative of monocyte-to-macrophage differentiation in each MonoMac cluster in MC38 tumors of D^WT^ and D^KO^ mice (pooled data; *n=4* per group). (B) Left: relative pseudotime along the monocyte-macrophage differentiation trajectory, inferred by Monocle. The arrow indicates the direction of pseudotime. States (“branches”) in the trajectory are indicated by numbers. Right: UMAP Projection of relative pseudotime for monocyte-macrophage differentiation onto the UMAP of the MonoMac clusters. Data are from MC38 tumors of D^WT^ and D^KO^ mice (pooled data; *n=4* per group). The scale (0–1) indicates the pseudotime. (C) Distribution of each MonoMac cluster across the monocyte-macrophage differentiation trajectory inferred by Monocle, in MC38 tumors of D^WT^ and D^KO^ mice (pooled data; *n=4* per group). Each color represents a Monomac cluster. (D) Left: density of cells in each MonoMac cluster across the monocyte-macrophage differentiation trajectory, inferred by Monocle. Concentric lines indicate cell density levels. Right: stacked barplot showing the number of cells from MonoMac clusters across states of the monocyte-macrophage differentiation trajectory inferred by Monocle. Data are from MC38 tumors of D^WT^ and D^KO^ mice (pooled data; *n=4* per group). (E) Top over- (red) or under-represented (blue) Hallmark pathways in states 1 (left) and state 5 (right) of the inferred Monocle trajectory. The number of downregulated (negative values) or upregulated (positive values) genes is indicated on the x axis. Over-representation analysis (ORA) was computed from the sets of state-specific enriched genes. *p* values adjusted for multiple testing according to the Benjamini-Hochberg method. (F) Enrichment scores in MonoMac clusters of MC38 tumors of D^WT^ and D^KO^ mice (pooled data; *n=4* per group) based on an M2-like macrophage signature derived from an *in vivo* dataset,^22^ plotted along the monocyte-macrophage differentiation trajectory inferred by Monocle. Numbers indicate states in the trajectory. (G) Top: density of cells in each MonoMac cluster, relative to monocyte-macrophage differentiation pseudotime. Bottom: heatmap showing the score of cell-cycle phases, M1/M2-like macrophage phenotypes, and Hallmark IFNγ response gene-set signatures, relative to monocyte-macrophage differentiation pseudotime. M1/M2-like macrophage phenotype signatures were derived from an *in vivo* dataset.^22^ Colors indicate enrichment scores, after conditional means smoothing and zero-centering. Data are from MC38 tumors of D^WT^ and D^KO^ mice (pooled data; *n=4* per group).

TAMs can originate from either recruitment of bone-marrow-derived monocytes or self-renewal of tissue-resident macrophages.^30^^,^^31^ Because monocyte-derived macrophage numbers increase as tumors progress,^32^ we addressed monocyte-to-macrophage differentiation as a first putative trajectory. We assessed the expression of canonical markers of monocytes (*Ly6c2*) and mature macrophages (*Adgre1*, encoding F4/80, and *Cd68*) in the MonoMac subpopulations and found that *Ly6c2* was more highly expressed in proinflammatory/M1-like Mono_1, Macro_1, and Macro_3 than in cycling/M2-like Macro_5, Macro_6, Macro_2 clusters (Figure 4A). In contrast, mature macrophage markers were higher in cycling/M2-like than in inflammatory/M1-like MonoMac. Interestingly, Macro_1 and Macro_3 showed intermediate expression of canonical monocyte and macrophage markers between Mono_1 and cycling/M2-like MonoMacs, which could indicate a transitional state. Overall, these results suggest that MonoMac subpopulations involve different stages of monocyte-to-macrophage differentiation.

To interrogate trajectories in the MonoMacs, we applied the Monocle algorithm, a method that uses pseudotime analysis to study trajectories.^33^^,^^34^^,^^35^ Monocle inferred five cell states stemming from state 1 (input pseudotime 0), projecting into states 2–5 through a node (state 3) (Figure 4B). State 1 was mostly composed of Mono_1, Macro_1, and Macro_3 and exhibited enrichment in transcriptomic signatures of proinflammatory pathways. At the opposite end of the trajectory, state 5 mainly comprised Macro_5, Macro_6, and Macro_2 and showed enrichment in cell cycle genes, such as E2F targets and G2M checkpoints, and downregulation of the interferon signature (Figures 4C–4E, S6A, and S6B). We confirmed these trajectories using the Palantir method, which employs diffusion-based pseudotime analysis to model cellular differentiation pathways and infer potential cell fates in high-dimensional single-cell data.^36^ The resulting trajectories (Figures S6C and S6D) were consistent with those obtained using Monocle. We next embedded macrophage M1/M2 signatures^22^ in Monocle trajectories, and observed a switch from M1 to M2 along the trajectory (Figure 4F). Thus, trajectories of monocyte-to-macrophage differentiation are associated with acquisition of proliferative states and an M1-to-M2-like phenotype switch.

Since we observed that a significant fraction of M2-like MonoMacs (Macro_5, Macro_6 and, partly, Macro_2) upregulated cell cycle genes, we speculated that the cell cycle could determine trajectories within MonoMacs. To test this hypothesis, we assigned scores to each cell based on expression of genes that identify distinct cell-cycle phases and plotted the scores along pseudotime obtained from Monocle, as described previously.^33^^,^^37^ Macro_5 and Macro_6 (state 5) aligned with active G1/S/G2M phases of the cell cycle, whereas an important fraction of Mono_1 and Macro_3 aligned with G0 phases (Figure 4G). We also examined gene expression patterns along the trajectory to assess whether the cell cycle was associated with functional features of the MonoMac. Cells within the G0/G1 phase, which correspond to the beginning of the trajectory, upregulated IFNγ response signatures, but this effect was lost when the cells progressed along the cell cycle within trajectories. In line with this functional programming potentially mediated through IFNγ, we noted a gradual loss of M1-like and gain of M2-like signatures when MonoMacs entered the cell cycle. We obtained similar results by applying the DeepCycle algorithm, which uses RNA velocity to determine a cell cycle-driven trajectory^38^ (Figure S6E). These results suggest a link between commitment to the cell cycle and monocyte differentiation into M2-like macrophages, which may parallel the transition from an immunostimulatory to an immunosuppressive phenotype.

### D^KO^ interferes with MonoMac trajectories and stalls them in a cell cycle-arrested state

**Figure 5:**
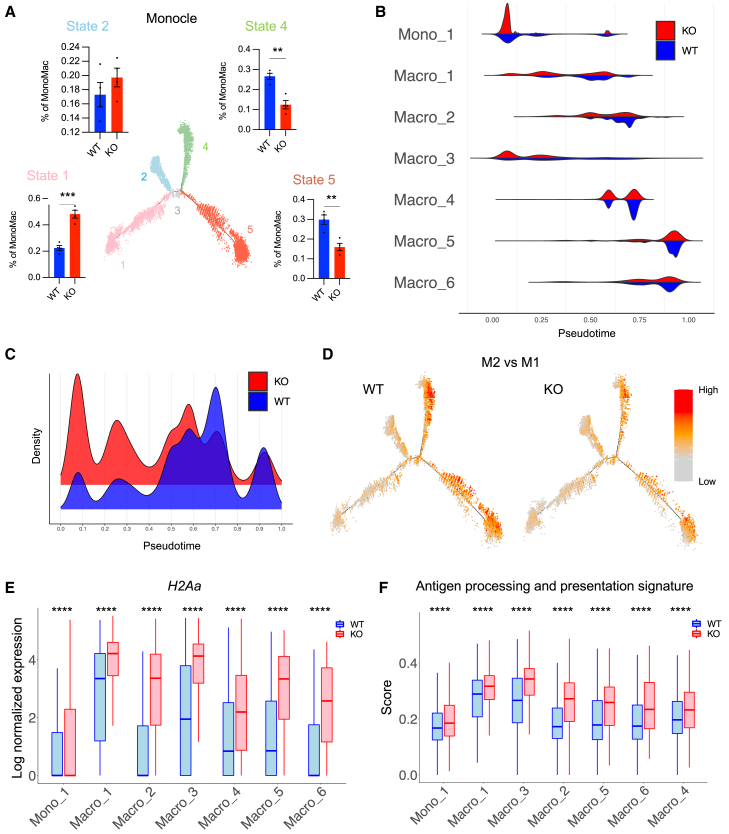
D^KO^ interferes with MonoMac trajectories and stalls them in a cell cycle-arrested state (A) Proportions of total MonoMac in states 1, 2, 4, and 5 of the monocyte-macrophage differentiation trajectory, inferred by Monocle by comparing tumors of D^WT^ and D^KO^ mice (*n=4*). Statistical analysis by unpaired Student’s t test. (B) Density of cells in each MonoMac cluster relative to monocyte-macrophage differentiation pseudotime, by comparing tumors of D^WT^ and D^KO^ mice (*n=4*). (C) Density of total MonoMac relative to monocyte-macrophage differentiation pseudotime, by comparing tumors of D^WT^ and D^KO^ mice (*n=4*). (D) Enrichment scores for the signature of M2-like macrophage phenotype (derived from an *in vivo* dataset^22^), by comparing tumors of D^WT^ and D^KO^ mice (*n=4*). Cells are plotted along the monocyte-macrophage differentiation trajectory inferred by Monocle. (E) Expression level of *H2-Aa* in each MonoMac cluster of tumors of D^WT^ and D^KO^ mice (*n=4*). Boxes represent the IQR, horizontal lines represent the median, and whiskers represent the range within 1.5 times the IQR. Statistical analysis by Student’s t test. (F) Enrichment score of the “antigen processing and presentation” gene ontology biological process (GOBP) term gene set in each MonoMac cluster of tumors of D^WT^ and D^KO^ mice (*n=4*). Boxes represent the IQR, horizontal lines represent the median, and whiskers represent the range within 1.5 times the IQR. Statistical analysis by Student’s t test. ∗∗*p* ≤ 0.01, ∗∗∗*p* ≤ 0.001, and ∗∗∗∗*p* ≤ 0.0001.

Having established that MonoMac heterogeneity is associated with trajectories from a cell cycle-arrested/M1-like to a cycling/M2-like state, we asked whether *Dicer1* deficiency interfered with this trajectory. Using Monocle, we compared D^KO^ and D^WT^ MonoMac states and found that their distribution in the trajectory was altered. In particular, D^KO^ skewed MonoMacs to a proinflammatory, M1-like and cell cycle-arrested state (Figures 5A–5D, S7A, and S7B), thereby restraining their maturation toward immunosuppressive M2-like macrophages. Accordingly, all MonoMac subpopulations upregulated *H2Aa* gene expression and antigen presentation signatures in D^KO^ tumors (Figures 5E and 5F).

We then validated some of these findings by flow cytometry of MC38 tumors from the cohorts illustrated in Figures 1A, S1A and S1B. We used Ly6C (inflammatory monocyte marker) and F4/80 (macrophage marker) to distinguish between immature monocytes, intermediate monocytes/macrophages, and mature macrophages, as previously described.^39^^,^^40^^,^^41^ We identified 3 monocyte/macrophage populations fairly corresponding to Mono_1 (Ly6C^+^F4/80^–^), Macro_1 plus Macro_3 (Ly6C^+^F4/80^+^), and Macro_2 plus Macro_4, Macro_5, and Macro_6 (Ly6C^−^F4/80^+^) (Figure S7C). In agreement with the scRNA-seq data, the mean fluorescence intensity (MFI) of Ly6C was highest in Mono_1-like cells and lowest in Macro_2,4,5,6-like cells, whereas that of F4/80 was highest in Macro_2,4,5,6-like cells and lowest in Mono_1-like cells. Of note, Ly6C was upregulated in Mono_1-like cells and Macro_1, 3-like cells, whereas F4/80 was upregulated in Macro_2,4,5,6-like cells, of D^KO^ compared to D^WT^ tumors (Figures S7D and S7E).

Together, these results indicate that D^KO^ affects MonoMac trajectories, causing them to stall in a cell cycle-arrested and proinflammatory state.

### D^KO^ restrains protumoral neutrophil differentiation

To explore the effects of D^KO^ macrophages on other cell types in the TME, we examined trajectories of neutrophil differentiation according to published results.^42^ Monocle inferred trajectories of immature (T1) and intermediate (T2) states transitioning to a terminally differentiated, proangiogenic and protumoral (T3) state in MC38 tumors (Figures S8A–S8C). Notably, macrophage D^KO^ partly restrained neutrophils from differentiating into the protumoral T3 state (Figures S8D and S8E). While (re)programming of neutrophil differentiation was likely macrophage-dependent, it cannot be formally excluded that *LysM-Cre*–mediated *Dicer1* inactivation also had cell-autonomous effects in the neutrophils that concurred to generate an antitumoral TME in D^KO^ mice.

### CD8^+^ T cell-derived IFNγ enriches M1-like MonoMacs in D^KO^ tumors

**Figure 6:**
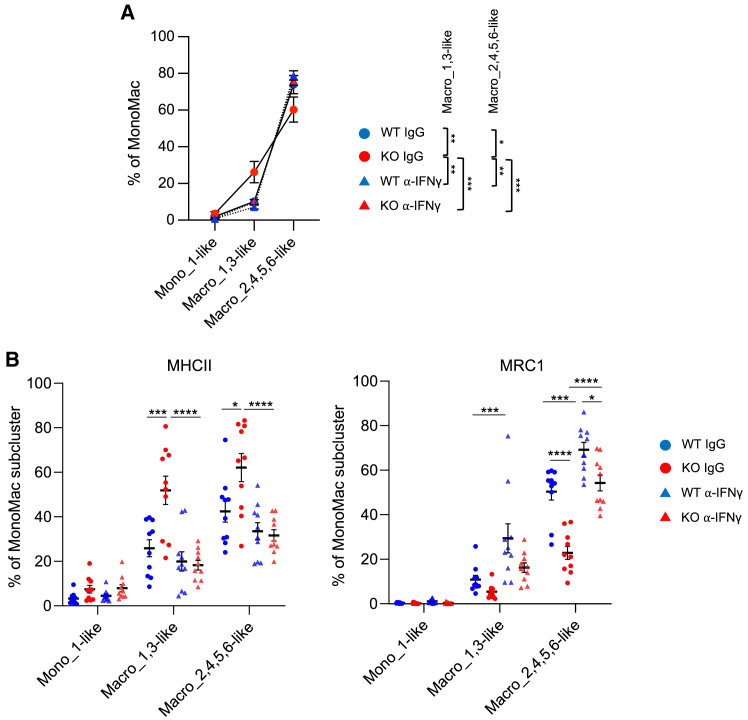
CD8^+^ T cell-derived IFNγ enriches M1-like MonoMacs in D^KO^ tumors (A) Percentage of the indicated monocyte/macrophage populations in tumors of D^KO^ or D^WT^ mice (mean ± SEM; *n=10*) treated with an IFNγ-neutralizing antibody (α-IFNγ) or control IgGs, analyzed by flow cytometry. Statistical analysis by two-way ANOVA with Sidak’s multiple comparison test. (B) Percentage of the indicated monocyte/macrophage populations expressing MHCII (left) or MRC1/CD206 (right) in tumors of D^KO^ or D^WT^ mice (mean ± SEM; *n=10*) treated with α-IFNγ or control IgGs, analyzed by flow cytometry. Statistical analysis by two-way ANOVA with Sidak’s multiple comparison test. ∗*p* ≤ 0.05, ∗∗*p* ≤ 0.01, ∗∗∗*p* ≤ 0.001, and ∗∗∗∗*p* ≤ 0.0001.

We next hypothesized that CD8^+^ T cell-derived IFNγ could underpin the perturbation of MonoMac trajectories in D^KO^ mice. We treated MC38 tumor-bearing mice with irrelevant IgGs or an IFNγ neutralizing antibody and interrogated the distribution of Mono_1-like, Macro_1,3-like, and Macro_2,4,5,6-like cells by flow cytometry. IFNγ neutralization in D^KO^ mice restored the proportions of Macro_1,3-like and Macro_2,4,5,6-like to levels similar to those in tumors of D^WT^ mice (Figure 6A). We also assessed the expression of MHCII, a marker of M1-like macrophages, and MRC1/CD206, a marker of M2-like macrophages, in each monocyte/macrophage populations.^41^ Whereas MHCII was upregulated and MRC1 downregulated in Macro_1,3-like and Macro_2,4,5,6-like cells of D^KO^ tumors, both effects were negated by IFNγ neutralization (Figure 6B). These results indicate that IFNγ directs MonoMac trajectories.

### D^KO^ enhances tumor response to antiangiogenic immunotherapy

**Figure 7:**
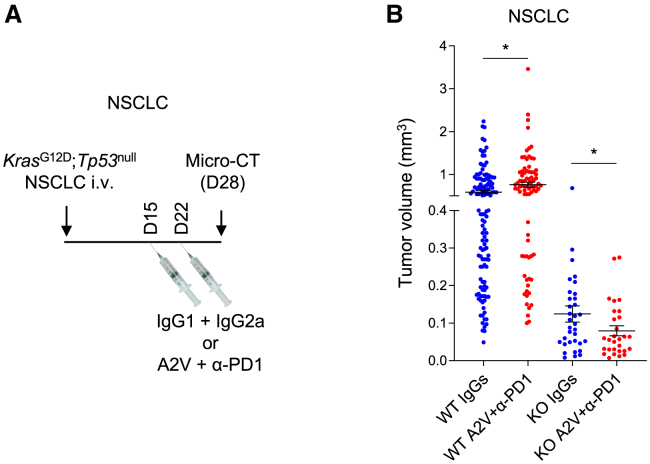
D^KO^ enhances tumor response to antiangiogenic immunotherapy (A) Schematic of the experimental setup. (B) Volume of individual lung tumors (mean ± SEM) in mice treated as indicated and imaged by micro-CT at day 28 post-tumor injection. *n=6* D^WT^ mice treated with IgG1+IgG2a (WT IgGs); *n=7* D^WT^ mice treated with A2V+α-PD1 (WT A2V+α-PD1); *n=5* D^KO^ mice (KO IgGs and KO A2V+α-PD1). Note that each mouse carries several independent lung tumors. Statistical analysis by unpaired Student’s t test performed between control and interventional treatment for each independent genotype. ∗*p* ≤ 0.05.

TAMs limit tumor response to immunotherapy or its combination with other agents, such as antiangiogenic drugs,^43^ in several cancer types including lung cancer.^5^^,^^6^^,^^7^^,^^8^ Depleting both monocyte-derived and lung-resident TAMs enhanced tumor response to “antiangiogenic immunotherapy” (a combination of VEGFA, angiopoietin-2, and PD1 blockade) in NSCLC models.^13^ We evaluated whether macrophage reprogramming in D^KO^ mice could phenocopy pharmacological TAM targeting and improve tumor response to antiangiogenic immunotherapy in the orthotopic *Kras*^G12D^;*Tp53*^null^ NSCLC model. To this aim, we administered A2V (a bispecific antibody against angiopoietin-2 and VEGFA^44^) and PD1 antibodies, or irrelevant IgGs, to mice 15 days post-tumor inoculation, and examined lung tumor growth at day 28 using micro-CT imaging. Consistent with our previous findings,^13^ combined A2V and anti-PD1 treatment not only failed to inhibit tumor growth but also led to accelerated progression of some tumors in D^WT^ mice (Figures 7A and 7B). Tumor growth was markedly inhibited in D^KO^ mice, and A2V plus PD1 blockade enhanced this effect. These results demonstrate that reprogramming TAMs to a proinflammatory/M1-like phenotype improves the efficacy of antiangiogenic immunotherapy in an experimental lung cancer model.

### Cycling/M2-like macrophages are conserved in human cancer

We asked whether the aforementioned MonoMac states and trajectories, which indicate transitions from a cell cycle-arrested, inflammatory/M1-like state to a proliferating, protumoral/M2-like state, are also present in human cancers. To this aim, we analyzed a publicly available dataset of several human cancer types.^45^

**Figure 8:**
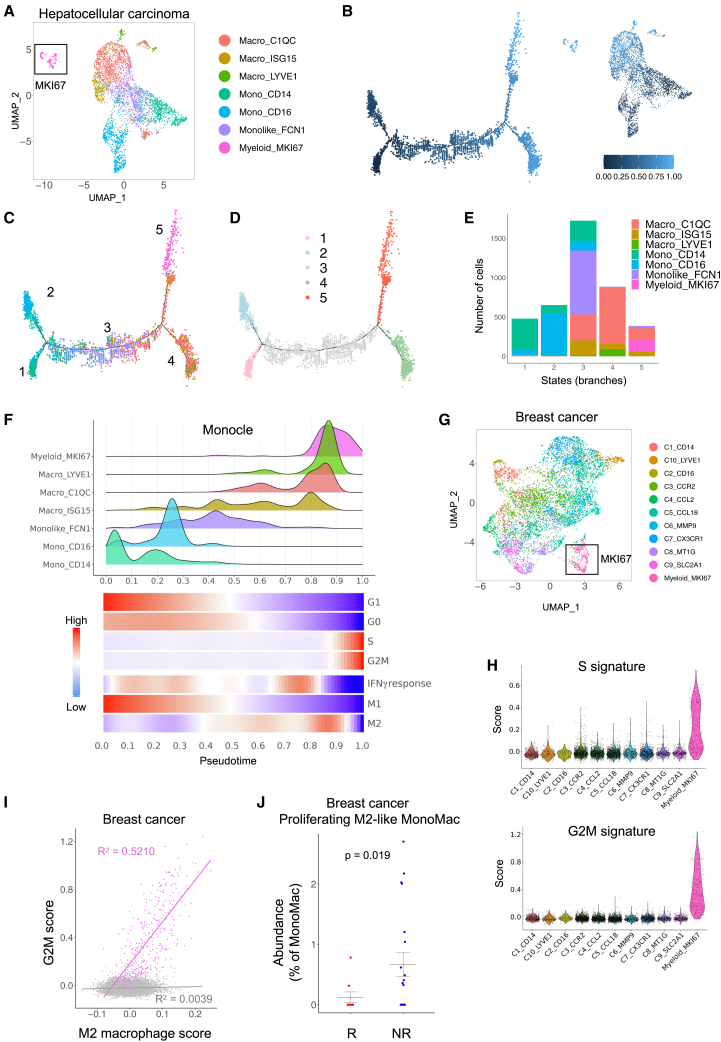
Cycling/M2-like macrophages are conserved in human cancer (A) UMAP projection of MonoMac in the human HCC dataset.^45^ Cells are color-coded according to subpopulation. Cycling macrophages (“Myeloid_MKI67”) are boxed. (B) Left: relative pseudotime along the monocyte-macrophage differentiation trajectory, inferred by Monocle. Right: UMAP representing relative pseudotime in the MonoMac cluster in the human HCC dataset. The scale (0–1) represents pseudotime. (C) Distribution of cells from each MonoMac cluster along the monocyte-macrophage differentiation trajectory, inferred by Monocle, in the human HCC dataset. Numbers indicate states in the trajectory D. Inferred trajectory states (“branches”) in the human HCC dataset. (E) Absolute frequencies (number of cells; stacked barplot) of clusters across states (“branches”) of the inferred Monocle trajectory in the human HCC dataset. Colors indicate MonoMac clusters. (F) Top: plot representing the density of cells in each MonoMac cluster from the human HCC dataset relative to monocyte-macrophage differentiation pseudotime. Bottom: heatmap showing the score of cell-cycle phases, M1/M2-like macrophage phenotypes, and Hallmark IFNγ response gene-set signatures, relative to monocyte-macrophage differentiation pseudotime. M1/M2-like macrophage phenotype signatures were derived from an *in vivo* dataset.^22^ Colors indicate enrichment scores, after conditional means smoothing and zero-centering. (G) UMAP projection of MonoMac from the human breast cancer dataset.^46^ Cells are color coded according to subpopulation. Cycling macrophages (“Myeloid_MKI67”) are boxed. (H) Scores of the S and G2M cell cycle phase signatures across MonoMac subpopulations from the human breast cancer dataset. Cells are color coded by subpopulation as in (G). (I) Correlation between an M2 macrophage signature score (derived from an *in vitro* dataset^21^) and a G2M cell-cycle score in cycling MonoMac (pink; adjusted R-squared = 0.5210; *p* ≤ 0.0001) but not other MonoMac subpopulations (gray; adjusted R-squared = 0.0039, *p* ≤ 0.0001). (J) Percentage (mean ± SEM) of cycling M2-like MonoMacs in responders (R, patients with clonal expansion of T cells; *n=9*) and non-responders (NR, patients without clonal expansion of T cells; *n=20*) to immunotherapy, based on the human breast cancer dataset.^46^ Statistical analysis by Student’s t test.

In human hepatocellular carcinoma (HCC),^45^ we identified a distinct cluster of cycling macrophages, called Myeloid_MKI67 (Figure 8A). In line with our findings in mice, Monocle revealed trajectories of monocyte-to-macrophage differentiation through five states (Figures 8B–8E). While monocytes (Mono_CD14 and Mono_CD16) were enriched at the beginning of the trajectory (states 1 and 2), Myeloid_MKI67 were present at the opposite end of the trajectory (state 5). Other monocyte/macrophage clusters (Monolike_FCN1, Macro_C1QC, Macro_ISG15, and Macro_LYVE1) identified intermediate states (2–4). In addition, the trajectory was associated with enrichment in S and G2M cell-cycle scores (and downregulation of G0/G1 scores), upregulation of the M2 macrophage score, and downregulation of the IFNγ response signature score (Figure 8F). We obtained similar results in human melanoma^45^ (Figures S9A–S9E).

We further confirmed the presence of myeloid_MKI67 cells in a dataset collating hormone receptor-positive and triple-negative breast cancers from patients who received neoadjuvant therapy with anti-PD1 antibodies^46^ (Figure 8G). Similar to HCC and melanoma, Myeloid_MKI67 cells were enriched in S and G2M cell-cycle scores (Figure 8H). In addition, there was a correlation between the M2 macrophage score and G2M score in Myeloid_MKI67 cells (Figure 8I). Notably, enrichment in proliferating, M2-like Myeloid_MKI67 correlated with a worse response to treatment (Figure 8J).

Together, these results show that trajectories from a cell cycle-arrested/M1-like to a cycling/M2-like macrophage state can be identified in certain human cancers and are associated with a worse response to immunotherapy in patients with breast cancer.

## Discussion

In this study, we employed mice with myeloid-specific *Dicer1* deletion (D^KO^) to investigate the cellular and molecular landscape of the TME in the context of enforced M1-like macrophage programming. Consistent with previous findings in mouse models of primary breast cancer, colon cancer, and glioblastoma,^11^^,^^12^ we observed a tumor-protective role of *Dicer1*-deficient, M1-like macrophages in both the MC38 colon cancer model and an orthotopic NSCLC model. In both settings, M1-like macrophage programming was associated with enhanced infiltration and activation of cytotoxic CD8^+^ T cells and marked inhibition of tumor growth, attesting to the pivotal role played by polarized macrophages in sculpting tumor progression.^5^^,^^6^^,^^7^^,^^8^ Importantly, M1-like macrophage programming in D^KO^ mice enhanced tumor response to a combination of antiangiogenic and immune therapy^43^ in a treatment-resistant model of experimental NSCLC.^13^

scRNA-seq of MC38 tumor-bearing mice revealed distinct cell clusters (or subpopulations) within the monocyte/macrophage (“MonoMac”) population, regardless of *Dicer1* genotype status. These clusters were further categorized into three main subgroups: inflammatory, hypoxic, and cycling/proliferative MonoMacs. Bioinformatic analysis of potential trajectories within the MonoMac population suggested a dynamic process of monocyte differentiation toward a bona fide macrophage state, which coincided with loss of M1-like immunostimulatory phenotypes and acquisition of M2-like immunosuppressive phenotypes. Further analyses indicated that these trajectories are embedded within the phases of the cell cycle. Indeed, cells at the beginning of the trajectory display a cell cycle-arrested phenotype, while progression along the trajectory leads to either a cycling/proliferative sub-state or a quiescent and hypoxic state. Of note, cycling M2-like macrophages were observed previously in mouse mammary tumor models.^47^ Interestingly, deletion of *Dicer1* in MonoMacs skewed theses trajectories and stalled monocyte differentiation at the beginning of the trajectory in the cell-cycle–arrested state associated with an M1-like, immunostimulatory state. These results align with a study showing that *Dicer1* inactivation in myeloid cells limits the proliferative capacity while enhancing the anti-tumoral functions of TAMs in a mouse glioblastoma model.^12^

IFNγ, which is highly expressed in exhausted CD8^+^ T cells, emerged as a pivotal orchestrator of immunostimulatory programming of the TME. Unsupervised clustering of genes and pathways that were significantly deregulated in tumors of D^KO^ mice revealed upregulation of several interferon-associated processes, such as antigen presentation, T cell activation, and response to interferons. Pharmacological IFNγ neutralization disrupted monocyte-to-macrophage differentiation, underscoring the impact of IFNγ in driving macrophage polarization toward an M1-like/antitumoral phenotype.

Cycling MonoMacs with an M2-like phenotype were conserved in human HCC and melanoma and were associated with a worse response to immunotherapy in human breast cancer. These findings should encourage investigating strategies for selectively targeting (or reprogramming) this cycling TAM subset in human cancer. We previously reported that cisplatin-mediated elimination of proliferative alveolar TAMs, which sustain Tregs, improved tumor response to antiangiogenic immunotherapy in a transgenic NSCLC model.^13^ Because cisplatin does not specifically target immunosuppressive and cycling TAMs, alternative strategies should be explored for selectively targeting these protumoral cells while sparing or enhancing immunostimulatory, M1-like TAMs, for example, through macrophage-targeted DICER inhibitors.^48^

### Limitations of the study

Our study has several limitations. The genetic model used to inactivate *Dicer1* in macrophages may also target other myeloid cells, such as neutrophils. We previously showed that the abundance of cellular miRNAs was not altered in neutrophils of D^KO^ mice.^11^ Indeed, while the *LysM*-Cre transgene is active in both macrophage and granulocyte-lineage cells, the short half-life of neutrophils^49^ may not allow for the decay of mature miRNAs ensuing upon disruption of DICER activity. In spite of this, we here observed altered neutrophil phenotypes in tumors of D^KO^ mice. This can be best interpreted as an indirect effect of macrophage reprogramming or IFNγ signaling on neutrophils,^50^^,^^51^ although cell-autonomous effects due to *Dicer1* inactivation and altered miRNA activity in neutrophils cannot be formally excluded.

The subcutaneous tumor model employed to study macrophage differentiation trajectories may underrepresent certain macrophage components found in spontaneously arising tumors. Indeed, whereas the vast majority of TAMs recruited to subcutaneously growing tumors are arguably of monocyte origin,^41^^,^^52^^,^^53^ those recruited to autochthonous tumors involve both monocyte-derived and locally derived, tissue-resident macrophages.^53^^,^^54^ Nevertheless, we confirmed our key findings in selected human cancers, in which differentiation trajectories encompassed monocytes on the one hand and cycling/M2-like macrophages on the other. We further identified potential associations between cycling/M2-like macrophages and response to neoadjuvant anti-PD1 therapy in breast cancer, but this clinical result warrants confirmation in additional cohorts of patients with cancer.

Finally, the potential influence of sex on the results was not considered in our study. For example, the MC38 tumor scRNA-seq study only used male mice, whereas the human breast cancer scRNA-seq analysis only involved female patients. Thus, sex-dependent effects on macrophage dynamics cannot be ruled out.

## Resource availability

### Lead contact

Further information and requests for resources and reagents should be directed to and will be fulfilled by the lead contact, Michele De Palma (michele.depalma@epfl.ch).

### Materials availability

All mouse genetic models generated in this study are available from the lead contact.

### Data and code availability

• Data. The scRNA-seq data generated in this study (mouse MC38 tumors) have been deposited in the Gene Expression Omnibus (GEO) and are publicly available as of the date of publication. The accession number is listed in the key resources table. Additional scRNA-seq data (human HCC and melanoma samples^45^) were publicly available in GEO and their accession number is listed in the key resources table. The human breast cancer scRNA-seq data^46^ were publicly available through the Diether Lambrechts Lab repository website (https://lambrechtslab.sites.vib.be/en/single-cell).
• Code. This paper does not report original codes.
• Any additional information required to reanalyze the data reported in this paper is available from the lead contact upon request.

## Acknowledgments

We thank Martina Schmittnaegel (Roche, Penzberg, Germany) for providing the A2V antibody. This study was supported by grants from the 10.13039/501100017035ISREC Foundation (Tandem to M.D.P. and N.M.), the 10.13039/501100001711Swiss National Science Foundation (SNSF 31003A-165963 and 310030-188868 to M.D.P., and CRSK-3_190441 to N.M.), and the 10.13039/501100000781European Research Council (ERC CoG EVOLVE-725051 to M.D.P.). The authors acknowledge the excellent technical support of the scientific platforms at EPFL and the Agora Cancer Center.

## Author contributions

F.D. performed experiments, analyzed and interpreted the data, and drafted the manuscript; J.L. conducted and N.F. supervised bioinformatics analyses; M.H., G.B., A.G., and C.W.-R. contributed to the execution of some experiments and to data analysis; N.M. and M.D.P. co-supervised the study, interpreted the data, and wrote the manuscript.

## Declaration of interests

None of the authors has competing interests related to this study. In the interest of full disclosure, M.D.P. has received sponsored research grants from EVIR Therapeutics, Hoffmann La-Roche and Deciphera Pharmaceuticals, serves on the Scientific Advisory Boards of EVIR Therapeutics, Montis Biosciences, Macomics, Deciphera Pharmaceuticals, Light Chain Bioscience/Novimmune, and Genenta and is an inventor on patents on engineered dendritic cells filed by EPFL.

## STAR★Methods

### Key resources table

**Table:** 

REAGENT or RESOURCE	SOURCE	IDENTIFIER
**Antibodies**		

Rat anti-CD16/CD32 (Fc block); clone 2.4G2	BD Biosciences	Cat#553142 RRID:AB_394656
Rat anti-CD45-APC-EF780; clone 30-F11	eBioscience	Cat#47-0451-82 RRID:AB_1548781
Rat anti-CD45-APC/Cy7; clone 30-F11	Biolegend	Cat#103115 RRID:AB_312980
Rat anti-CD45-PE; clone 30-F11	Biolegend	Cat#103105 RRID:AB_312970
Rat anti-CD11b-BV711; clone M1/70	Biolegend	Cat#101241 RRID:AB_2563310
Rat anti-Ly6C-AF700; clone HK1.4	Biolegend	Cat#128024 RRID:AB_10643270
Rat anti-Ly6C-BV605, clone HK1.4	Biolegend	Cat#128035 RRID:AB_2562352
Rat anti-Ly6G-PB; clone 1A8	Biolegend	Cat#127611 RRID:AB_1877212
Rat Ly6G-PerCP-Cy5.5; clone 1A8	Biolegend	Cat#127615 RRID:AB_1877271
Rat anti-F4/80-FITC; clone BM8	Biolegend	Cat#123107 RRID:AB_893500
Rat anti-F4/80-AF488; clone BM8	Biolegend	Cat#123119 RRID:AB_893479
Rat anti-MRC1-AF647; clone C068C2	Biolegend	Cat#141717 RRID:AB_2562232
Rat anti-MRC1-APC; clone C068C2	Biolegend	Cat#141707 RRID:AB_10896057
Rat anti-MHCII-PerCP-Cy5.5; clone M5/114.15.2	Biolegend	Cat#107625 RRID:AB_2191071
Rat anti-CD3-BV605; clone 17A2	Biolegend	Cat#100237 RRID:AB_2562039
Rat anti-CD8α-AF647; clone 53-6.7	BD Bioscience	Cat#557682 RRID:AB_396792
Rat anti-CD8α-PE; clone YTS169.4	ThermoFisher Scientific	Cat#MA1-82111 RRID:AB_2075775
Rat anti-CD4-PE; clone RM4-5	Biolegend	Cat#100512 RRID:AB_312714
Rat anti-CD4-Pacific Blue; clone RM4-5	Biolegend	Cat#100531 RRID:AB_493374
Murinized anti-VEGFA/ANGPT2 (A2V) IgG2a	Roche	N/A
Rat IgG2a anti–PD1; clone RMPI-14	BioXCell	Cat#BE0146 RRID:AB_10949053
Rat IgG1 anti-mouse IFNγ; clone XMG1.2	BioXCell	Cat#BE0055 RRID:AB_1107694
Rat IgG2a Isotype Control; clone 2A3	BioXCell	Cat#BE0089 RRID:AB_1107769
Murinized IgG1; clone MOPC-21	Roche	N/A

**Chemicals, peptides, and recombinant proteins**		

Iscove’s modified Dulbecco’s medium (IMDM)	Gibco	Cat#12440-053
Fetal bovine serum (FBS)	Gibco	Cat#10437-028
L-glutamine	Gibco	Cat#25030-024
Penicillin/streptomycin	Gibco	Cat#15140-122
Collagenase IV	Stemcell Technologies Switzerland GmbH	Cat#100-0680
Trypsin-EDTA (0.05%), phenol red-	Life Technologies EU BV	Cat#100-0680
Dispase II, powder	Life Technologies	Cat#17105041
DNase I	Sigma-Aldrich Chemie GmbH	Cat#10104159001
EDTA	Invitrogen	Cat#15575-020
Cryomatrix	Histocom AG	Cat#6769006(HIS)
Methanol	VWR International GmbH	Cat#1.07018.2511
Bovine serum albumin	Sigma-Aldrich Chemie GmbH	Cat#A7906-100G
DAPI	Sigma-Aldrich	Cat#D9542-1MG
Dako fluorescence mounting medium	Agilent Technologies Schweiz AG	Cat#S302380-2
Chloroform	Sigma-Aldrich	Cat#67-66-3

**Critical commercial assays**		

Mouse IFNγ ELISA Kit	STEMCELL Technologies	Cat#02020
RNeasy Mini Kit	Qiagen	Cat#74104
SuperScript Vilo cDNA Synthesis Kit	Invitrogen	Cat#11754250
TaqMan™ Universal PCR Master Mix	Applied Biosystems	Cat#4444557
Single cell RNA seq Chromium 10X kit	10x Chromium	

**Deposited data**		

scRNA-seq data of mouse MC38 tumors	This study	GEO: GSE274402
scRNA-seq data of human hepatocellular carcinoma and melanoma	Cheng et al.^45^	GEO: GSE154763
scRNA-seq data of human breast cancer	Bassez et al.^46^	https://lambrechtslab.sites.vib.be/en/single-cell

**Experimental models: Cell lines**		

MC38 colon adenocarcinoma cell line (derived from C57BL6 mouse)	Baer et al.^11^	
KP *(Kras*^G12D^;*Tp53*^null^) lung cancer cell line (derived from C57BL6 mouse)	Martinez-Usatorre^13^	

**Experimental models: Organisms/strains**		

LyzM Cre x Dicer1lox mice	Baer et al.^11^	
C57BL6J	JAX	JAX:000664

**Software and algorithms**		

Prism	GraphPad Software	
Micro-CT imaging analysis	Osirix-MD (Pixmeo)	
Cell Ranger Single Cell Software Suite	10x Genomics	v. 3.1.0
R	https://www.R-project.org/	v. 4.1.1
Seurat	CRAN	v. 4.0.4
ggpubr	CRAN	v. 0.4.0
muscat	Bioconductor	v. 1.16.0
clusterProfiler	Bioconductor	v. 4.0.5
msigdbr	Bioconductor	v. 7.4.1
ProjecTILs	https://github.com/carmonalab/ProjecTILs	v. 2.0.3
UCell	https://github.com/carmonalab/UCell	v. 1.3.1
Monocle	Bioconductor	v. 2.20.0
MAST	Bioconductor	v. 1.18.0
DDRTree	CRAN	v. 0.1.5
Palantir	https://github.com/dpeerlab/Palantir	v. 2.1.1
Python	https://www.python.org/	v. 3.11.6
DeepCycle	https://github.com/theislab/deepcycle	
Velocyto	https://velocyto.org/	v. 0.17
scVelo	https://scvelo.readthedocs.io	v. 0.2.4
destiny	Bioconductor	v. 3.20
STACAS	https://github.com/carmonalab/STACAS	v. 2.2.2

### Experimental model and study participant details

#### Cell lines

Mouse MC38 colon adenocarcinoma cells were available in the De Palma laboratory (previously obtained from P. Romero, University of Lausanne, Switzerland). Mouse KP *(Kras*^G12D^;*Tp53*^null^) lung cancer cells were available in the De Palma laboratory (previously obtained from E. Meylan, EPFL, Switzerland). Both MC38 and KP cells were maintained in Iscove’s modified Dulbecco’s medium (IMDM) supplemented with 10% fetal bovine serum (FBS), 2 mM L-glutamine and a combination of 50 units ml^−1^ penicillin and 50 μg ml^−1^ streptomycin. No cell lines used in this study were found in databases of commonly misidentified cell lines. The cell lines have not been authenticated recently but have been routinely used in the laboratory, demonstrating the expected properties and behaviour when inoculated in mice. All cell lines regularly tested negative for *Mycoplasma* contamination.

#### Mice

For MC38 tumor studies, *Dicer1*^lox/lox^ mice (C57BL/6 background) were crossed with C57BL/6/*LysM*-Cre mice (C57BL/6 background) to obtain *LysM*-Cre^+/WT^;*Dicer*^–/–^ mice. Although each experiment used mice of one sex, both male and female mice, aged 5 to 10 weeks, were used in different MC38 experiments. The scRNA-seq study used male mice. For the orthotopic NSCLC studies, *Dicer1*^lox/lox^ mice were crossed with C57BL/6/*LysM*-Cre mice to obtain *LysM*-Cre^+/+^;*Dicer*^–/–^ mice. Only male mice, aged 7 to 10 weeks, were used in the orthotopic NSCLC experiments.

Pups were genotyped by Transnetyx using qPCR assays, as detailed on the company’s website (http://www.transnetyx.com). All mice used in this study were maintained in the pathogen-free barrier animal facility of EPFL, adhering to Swiss regulations for the care and use of mice in experimental research. Mice were housed in groups of up to five mice per cage at 18-24 °C with 40–60% humidity and maintained on a 12:12 h light:dark cycle (06:00-18:00 h). All procedures were performed in accordance with protocols approved by the veterinary authorities of the Canton Vaud under Swiss law (VD2916; VD3435; VD2978).

#### Subcutaneous MC38 model

MC38 cells were passaged at least three times to obtain actively growing cells. The cells were then resuspended in PBS at a concentration of 10 × 10ˆ6 cells per ml, and 100 μl of the cell suspension was injected subcutaneously into the right flank of the mice. The tumor size was measured using calipers, calculating the volume with the formula: tumor volume = ½ × d^2^ × D, where D is the long diameter and d is the short diameter.

#### Orthotopic NSCLC model

Confluent *Kras*^G12D^;*Tp53*^null^ NSCLC cells, established as described previously,^13^ were detached using trypsin to create single-cell suspensions. The cells were then cultured in 30 μl droplets of complete medium for 12 hours, allowing them to aggregate. Five droplets were then injected directly into the tail vein of the mice. Tumors were imaged and measured by micro-CT imaging and volumetric analysis.

#### MC38 tumor scRNA-seq datasets

We conducted scRNA-seq of MC38 tumors grown in either D^WT^ or D^KO^ mice (n=4 per group) for 11-12 days. Only male mice were used.

#### Human tumor scRNA-seq datasets

We re-analyzed publicly available human scRNA-seq datasets.^45^^,^^46^ The HCC dataset comprised samples from 5 patients.^45^ The melanoma dataset comprised samples from 16 patients,^45^ all shown in the UMAP of Figure S9A, but 2 samples with less than 100 cells each were not used for the trajectory analyses. The breast cancer dataset comprised samples from 29 patients, of which 9 were classified as responders and 20 as non-responders.^46^ Detailed information on the patients characteristics is available in the original publications.^45^^,^^46^

### Method details

#### Treatment of mice with neutralizing antibodies

The IFNγ-neutralizing antibody (12 mg/kg; clone XMG1.2, rat IgG1, Bio X Cell) was administered intraperitoneally twice per week starting from day 7 after MC38 tumor injection, until the end of the experiment. The anti-VEGFA/ANGPT2 (A2V; 20 mg/kg; murinized IgG2a, Roche) and anti-PD1 (10 mg/kg; rat IgG2a, clone RMPI-14, BE0146, Bio X Cell) antibodies were administered on days 15 and 22 post-NSCLC injection. For the latter study, irrelevant control IgGs were a combination of IgG1 (20 mg/kg; clone MOPC-21, Roche) and rat IgG2a (10 mg/kg; clone 2A3, BE0089, Bio X Cell).

#### Tumor monitoring by micro-CT

Micro-CT scans of the lungs of NSCLC–bearing mice were obtained using a Quantum FX micro-CT scanner (PerkinElmer). Mice were anesthetized with isoflurane throughout the imaging procedure. Imaging was performed once a week, starting from day 12 after tumor injection.

#### Analysis of micro-CT scans

For measuring tumor volume (volumetry), micro-CT scans were processed and analyzed using Analyze software. 3D reconstruction was performed using Osirix-MD (Pixmeo). Tumors appeared as solid masses with a ground-glass opacity and a spherical shape. The pixel intensity range of the tumors was manually adjusted based on the contrast between the tumor and lung tissue to allow the software to automatically determine and trace the tumor margins. Manual adjustments were made when necessary. Individual tumors were defined as separate objects, and their volumes were calculated by the software.

#### Tissue processing

An intracardiac perfusion with PBS was performed before organ collection.

*Flow cytometry analysis*. Tumors were minced with scissors and digested in a mixture of collagenase IV (0.2 mg/ml, Worthington), dispase (2 mg/ml, Life Technologies), and DNase I (0.1 mg/ml, Life Technologies) diluted in IMDM medium (Sigma-Aldrich) for 20 minutes at 37°C with mixing. Cell suspensions were filtered through 70 μm cell strainers and washed with FACS buffer (2% FBS, 2mM EDTA in PBS) before antibody staining.

*qPCR analysis*. Tumors were harvested, snap-frozen in liquid nitrogen, and stored at –80°C.

*Immunofluorescence staining of lung tumors*. Tumors were dissected from the lung and embedded in O.C.T. compound (Cryomatrix, Thermo Fisher Scientific) and subsequently placed on dry ice. Blocks were stored at – 80°C. Tissue sections of 8 μm were obtained using a Leica cryostat CM1950 (Leica Biosystems) and stored at –20°C.

#### Immunofluorescence staining of lung tumors

For CD8 staining, lung tumor sections were first fixed in methanol for 20 minutes at –20°C, washed three times with PBS for 5 minutes each, and incubated with blocking solution containing 1% bovine serum albumin (BSA) and 5% FBS in PBS for 1 hour at room temperature. Sections were then incubated overnight at 4°C in 100-200 μL of blocking solution containing rat anti-CD8α-AF647 (1:50, clone 53-6.7, 557682, BD Bioscience). After staining, nuclei were labeled with DAPI (1 μg/ml, Sigma Aldrich) and sections mounted in Dako fluorescence mounting medium, covered with cover glass (Heathrow Scientific), and stored at 4°C. Slides were washed three times with PBS for 5 minutes between each step. Images were acquired using Zeiss LSM700 confocal microscope and analyzed with Image J without knowledge of the treatment group.

#### Flow cytometry

Tumor cell suspensions were incubated with Fc block (rat anti-CD16/CD32, 1:100, clone 2.4G2, 553142, BD Bioscience) before surface marker staining with combinations of the antibodies listed in the Key Resource Table and below:

CD45-APC-eF780 (1:200, clone 30-F11, 47-0451-82, ebioscience)

CD45-APC-Cy7 (1:200, clone 30-F11, 103115, BioLegend)

CD45-PE (1:400, clone 30-F11, 103105, BioLegend)

CD3-BV605 (1:200, clone 17A2, 100237, Biolegend)

CD4-PE (1:200, clone RM4-5, 100512, Biolegend)

CD4-PB (1:200, clone RM4-5, 100547, Biolegend)

CD8α-PE (1:50, clone YTS169.4, MA182111, ThermoFisher Scientific)

CD8α -AF647 (1:100, clone clone 53-6.7, BD Bioscience)

CD11b-BV711 (1:200, clone M1/70, 101241, Biolegend)

F4/80-AF488 (1:100, clone BM8, 123119, Biolegend)

F4/80-FITC (1:200, clone BM8, 123107, BioLegend)

MRC1-AF647 (1:100, clone C068C2, 141717, Biolegend)

MRC1-APC (1:100, clone C068C2, 141707, BioLegend)

MHCII-PerCP-Cy5.5 (1:400, clone M5/114.15.2, 107625, Biolegend)

Ly6C-AF700 (1:100, clone HK1.4, 128024, Biolegend)

Ly6C-BV605 (1:200, clone HK1.4, 128035, BioLegend)

Ly6G-PerCP-Cy5.5 (1:200, 1A8, 127615, Biolegend)

Ly6G-PB (1:400, clone 1A8, 127611, BioLegend)

Cell staining was performed in darkness at 4°C for 20-30 minutes in FACS buffer, except for LIVE/DEAD staining, which was performed in PBS. Cells were washed between each step with FACS buffer, PBS, and resuspended in FACS buffer before analysis with an LSRII SORP apparatus (BD Biosciences). Data were analyzed using FlowJo. Cells were gated based on their size and granularity (FSC-A versus SSC-A), followed by doublet and dead-cell exclusion.

Distinct CD45^+^ hematopoietic cell types were identified as follows:

CD3^+^ T cells: CD45^+^ CD11b^–^ CD3^+^

CD4^+^ T cells: CD45^+^ CD11b^–^ CD3^+^ CD8^–^ CD4^+^

CD8^+^ T cells: CD45^+^ CD11b^–^ CD3^+^ CD4^–^ CD8^+^

Neutrophils: CD45^+^ CD11b^+^ Ly6G^+^

Monocytes: CD45^+^ CD11b^+^ Ly6G^–^ F4/80^–^ Ly6C^+^

Tumor-associated macrophages (TAMs): CD45^+^ CD11b^+^ Ly6C^–^ Ly6G^–^ F4/80^+^

M2-like TAMs: CD45^+^ CD11b^+^ Ly6C^–^ Ly6G^–^ F4/80^+^ MRC1^+^

Mono_1-like cells: CD45^+^ CD11b^+^ Ly6G^–^ F4/80^–^ Ly6C^+^ (with and w/o MRC1 and MHCII)

Macro_1,3-like cells: CD45^+^ CD11b^+^ Ly6G^–^ F4/80^+^ Ly6C^+^ (with and w/o MRC1 and MHCII)

Macro_2,4,5,6-like cells: CD45^+^ CD11b^+^ Ly6G^–^ F4/80^+^ Ly6C^–^ (with and w/o MRC1 and MHCII)

#### IFNγ measurement by qPCR and ELISA

For RNA extraction and qPCR analysis, total mRNA was extracted using the RNeasy Mini Kit (Qiagen). Briefly, samples were homogenized in 700 μl of QIAzol lysis reagent, followed by addition of chloroform and centrifugation. The upper phase was transferred to a new tube, mixed with ethanol, and loaded onto RNeasy Mini columns. After washing steps with Buffer RWT and Buffer RPE, RNA was eluted in RNase-free water. RNA concentration was quantified using NanoDrop ND-2000. cDNA was synthesized using the SuperScript Vilo cDNA Synthesis Kit (Invitrogen). RNA (up to 20 μl) was mixed with 4 μl of 5x VILO Reaction Mix and 2 μl of 10x SuperScript Enzyme Mix. Reverse transcription was performed at 25°C for 10 min, 42°C for 60 min, and 85°C for 5 min. cDNA samples were stored at -20°C. TaqMan gene assays (Applied Biosystems/Life Technologies, *Ifng* probel Mm01168134_m1) were used. Each reaction contained 10 ng of cDNA per well in triplicate in a 384-well plate. A master mix was prepared with qPCR Universal master mix and TaqMan gene assay. qPCR was run for 40 cycles on an ABI7900HT apparatus. Raw data were analyzed using SDS software v2.4. Relative gene expression levels were calculated using the ΔΔCt method normalized to a reference gene (*Gapdh*).

Serum IFNγ was measured using the mouse IFNγ ELISA kit (StemCell Technologies 02020), following the manufacturer’s instructions.

#### Single-cell RNA sequencing (mouse MC38 tumors)

##### Sample preparation

Tumors were minced with scissors and digested in a mixture of collagenase IV (0.2 mg/ml, Worthington), dispase (2 mg/ml, Life Technologies), and DNase I (0.1 mg/ml, Life Technologies) diluted in IMDM medium (Sigma-Aldrich) for 20 minutes at 37°C with mixing. Single cells were filtrated through a 40 μm Flowmi strainer (Bel-Art) and resuspended in PBS with 0.04% BSA, checked for the absence of doublets or aggregates and loaded into a Chromium Single Cell Controller (10× Genomics, Pleasanton) in a chip together with beads, master mix reagents (containing reverse transcriptase and poly-dT primers) and oil to generate single-cell-containing droplets.

##### Data acquisition

Single-cell gene expression libraries were prepared using Chromium Single Cell 3’ Library & Gel Bead Kit v2 following the manufacturer's instruction. Quality control was performed with a TapeStation 4200 (Agilent) and QuBit dsDNA high sensitivity assay (Thermo fisher scientific) following manufacturer instructions. The libraries were sequenced on an Illumina HiSeq4000 platform, with run conditions as per 10X recommendations, aiming at 50’000 reads/cell.

##### Data processing

The Cell Ranger Single Cell Software Suite v3.1.0 was used to perform sample demultiplexing, barcode processing, and 3’ gene counting using 10X Genomics custom annotation of murine genome assembly mm10-3.0.0. Data were analyzed using the standard functions from the Seurat R package (v. 4.0.4).^55^ Breifly, cells above 1% and below 99% of total feature counts were kept. Cells with more than 10% of features mapping to the mitochondrial genome were discarded. Raw data was integrated, scaled and log-transformed; principal component analysis (PCA) was performed based on the expression of the top 2000 most variable genes, selected using the variance-stabilizing transformation (“vst”) method; Uniform Manifold Approximation and Projection (UMAP) dimensional reduction was performed based on the first 20 principal components. Unsupervised clustering was performed applying the graph-based clustering approach and Louvain algorithm at different resolutions, based previously computed neighbor graph using the top 30 PCs. Clusters were identified and merged into cell populations based on cell type specific markers (Table S1).

##### Differential gene expression and pathway enrichement analyses

Differential gene expression was computed within each cell population between conditions using the pseudo-bulk approach implemented in muscat (v1.16.0; https://github.com/HelenaLC/muscat), using the method edgeR, or using FindAllMarkers. Genes expressed in at least 10% of the cells per population per sample and with average logCPM > 5 were retained (Table S1). Gene set enrichment analysis was performed with clusterProfiler (v.4.0.5)^56^ applying GSEA with default parameters and 100’000 permutations to obtain P-values. Overrepresentation analysis was performed on a subset of regulated genes applying clusterProfiler enrichr. The Hallmark collection from the Molecular Signatures Database (MsigDB),^57^^,^^58^^,^^59^ obtained through the msigdbr R package (v.7.4.1),^58^ was used, or specific gene signatures extracted from published studies or in mSigDB. Differential abundance of cell populations between D^WT^ and D^KO^ mice was based on Student’s *t-*test for comparison of means. P-values were adjusted for multiple testing using the Benjamini-Hochberg method.

##### Prediction of T lymphocyte cellular states

The R package ProjecTILs (v 2.0.3) was used to project T lymphocyte transcriptomic profiles onto a reference map of tumor-infiltrating lymphocytes (TILs) cellular states.^15^ The pre-computed cross-study pan-cancer murine TIL Atlas version 1.0 (http://tilatlas.unil.ch/) was downloaded and used as the reference map. Prediction was performed using a nearest-neighbor classifier and was based on a majority vote of its annotated nearest neighbors in PCA space. The first ten principal components were used to compute distances, and the majority vote was based on the 20th nearest neighbors.

##### Cell cycle phase enrichment scores

G1, S and G2M cell cycle phases were scored using the CellCycleScoring function from the Seurat R package and the cell cycle phase markers from Tirosh et al*.*^37^ The G0 cell cycle phase signature was obtained from Rappez et al.^38^ and scored using the AddModuleScore_Ucell function from the Seurat R package (v. 4.0.4), which computes the Mann-Whitney U statistic implemented in the Ucell R package (v. 1.3.1).^60^

##### M1/M2-like signatures and IFNγ response enrichment scores

Tumor-associated macrophage signatures (M1-like and M2-like) were obtained from ref. 21 and 22. The signature for IFNγ response consisted of the Hallmark pathway “Interferon Gamma response” gene set, and was obtained from the MsigDB through the msigdbr R package. Signature enrichments were scored using the AddModuleScore_Ucell function from the Seurat R package.

##### Trajectory analysis of MonoMac subpopulations with Monocle

Trajectory analysis of MonoMac subpopulations was performed using functions from the Monocle R package (v. 2.20.0).^33^^,^^34^^,^^35^ Only cells expressing at least 500 genes, and genes expressed in at least 25% of cells that were also positive markers for the MonoMac subpopulations, were considered. Markers were identified using the FindAllMarkers^55^ function from the Seurat R package, using a threshold for the minimum average log2 fold change of 0.35. Differential expression was tested using the hurdle model implemented in the MAST R package (v. 1.18.0; https://github.com/RGLab/MAST/). The trajectory was learned by first reducing the dimensionality of the data (reduceDimension function) and then ordering cells according to a pseudotime (orderCells function). Dimensionality was reduced to two using the Discriminative Dimensionality Reduction with Trees algorithm implemented in the DDRTree R package (v. 0.1.5; https://github.com/cole-trapnell-lab/DDRTree). Cells were ordered without any pre-determined “root” state or number of end-point cell states. Positive markers for different states in the trajectory were determined using the FindAllMarkers function from the Seurat package, ranked according to log fold change, and used for performing over-representation analysis (ORA) of Hallmark pathways gene sets (obtained through the msigdbr R package). ORA was performed using the enricher function from the clusterProfiler R package (v. 4.0.5).^56^

##### Diffusion-based pseudotime analysis of MonoMac differentiation with Palantir

Diffusion-based pseudotime analysis of MonoMac differentiation was performed using the Palantir package (v. 2.1.1)^36^ in Python (v. 3.11.6). Initially, a low-dimensional phenotypic manifold of the expression data was constructed by creating a diffusion map based on the first 27 principal components, which collectively explained at least 85% of the variance in the data. Subsequently, Markov Affinity-based Graph Imputation of Cells (MAGIC) was applied to determine gene expression trends. The Palantir algorithm was then employed to align cells along differentiation trajectories and determine pseudotime. For the starting point, a cell from the Mono_1 cluster was selected based on the extreme value of diffusion component 1, which was associated with monocyte to macrophage differentiation.

##### Assignment of a continuous cell cycle trajectory to MonoMac cells

Cell cycle trajectory analysis of MonoMac cells was performed using DeepCycle.^61^ First, we used Velocyto (v. 0.17)^62^ to create loom files with read counts divided by spliced, unspliced or ambiguous mRNA. Loom files were then used to compute the moments for RNA velocity estimation using scVelo (v.0.2.4).^63^ Finally, the results from RNA velocity were used to run Deepcycle in two steps. In the first step, a random gene from the provided list of mouse cycling genes (GOterm:cell_cycle, GO:0007049) was used as the initial condition for the transcriptional phase, together with the hotelling filter and an expression threshold of 0.1. In the second step, Actb was chosen as the gene for the initial condition for the transcriptional phase, the hotelling filter was not used, and the expression threshold was set to 0.5.

##### Cell cycle phase enrichment scores

The G1, S and G2M cell cycle phases were scored using the CellCycleScoring function from the Seurat R package, and the cell cycle phase markers from Tirosh et al., 2015.^37^ The G0 cell cycle phase signature was obtained from Rappez et al., 2020^38^ and scored using the AddModuleScore_Ucell function.

##### M1/M2-like signatures and IFNγ response enrichment scores

Tumor-associated macrophage signatures (M1-like and M2-like) were obtained from published datasets.^21^^,^^22^ The signature for IFNγ response consisted of the Hallmark pathway “Interferon Gamma response” gene set, and was obtained from the MsigDB through the msigdbr R package. Signature enrichments were scored using the AddModuleScore_Ucell function from the Seurat R package.

##### Differential abundance of MonoMac subpopulations

The analysis of differential abundance of cell populations between D^WT^ and D^KO^ mice was based on Student’s *t*-test for comparison of means. P-values were adjusted for multiple testing using the Benjamini-Hochberg method.

##### Analysis of neutrophil differentiation

Neutrophil differentiation in the tumor microenvironment was analyzed using a diffusion map generated with the destiny R package (version 3.20).^64^ The map was constructed based on the integrated gene expression matrix of neutrophils, utilizing the first 50 principal components. Signatures for T1, T2, and T3 neutrophil states were derived from the heatmap presented in Figure 2F of ref. 42. Signature enrichments were quantified using the AddModuleScore_Ucell function from the Seurat R package. To compare signature scores between D^WT^ and D^KO^ mice, Student’s *t-*tests were employed. The resulting P-values were adjusted for multiple testing using the Benjamini-Hochberg method to control for false discovery rate.

#### Macrophage signatures in human cancer

The hepatocellular carcinoma and melanoma human scRNA-seq data were obtained from the Gene Expression Omnibus (GEO: GSE154763).^45^ Data analysis was conducted using standard functions from the Seurat R package (version 4.0.4). Raw data were log-normalized, scaled, and centered. Principal component analysis (PCA) was performed based on the expression of the top 2,000 most variable genes, selected using the variance-stabilizing transformation (VST) method. Semi-supervised integration of data from different patients was conducted using the STACAS R package (version 2.2.2).^65^ The integration process was based on the top 2,000 most variable genes and the first twenty principal components. To guide the integration, cell population annotations retrieved from the GEO database were utilized. Uniform Manifold Approximation and Projection (UMAP) dimensional reduction of the integrated data was applied using the first ten principal components. Cell cycle and tumor-associated macrophage signature scoring were conducted following the procedure described above.

The breast cancer human scRNA-seq data were downloaded from the Diether Lambrechts Lab website (http://biokey.lambrechtslab.org).^46^ Only samples from on-treatment surgical resections were selected for analysis. Data processing was performed using standard Seurat functions. Raw counts were log-normalized and scaled. PCA was conducted based on the expression of the top 2,000 variable features, and UMAP was performed on the first 20 principal components. A nearest-neighbor graph was constructed and utilized for unsupervised clustering of cells with a resolution of 0.5. The M2-like proliferating cluster of cells was identified based on *MKI67* expression, cell cycle phase scoring, and tumor-associated macrophage signature scoring, following the procedure described above.

Correlation analysis between G2M and M2-like signature scores in MKI67^+^ macrophages was performed using the cor.test function in R. The mean abundance of M2-like proliferating cells (as a proportion of total MonoMac population) per patient was compared between treatment responders and non-responders using a Student's *t*-test.

*Trajectory analysis of MonoMac differentiation in human cancer*. Trajectory analysis of MonoMac differentiation in human cancer was conducted using the Monocle R package. The analytical procedure followed the same methodology as described for the mouse data.

### Quantification and statistical analysis

#### Statistical analysis

Graphs were generated and statistical analyses performed using Prism (GraphPad Software) or functions from the ggpubr R package (v. 0.4.0).^66^ Error bars represent the standard error of the mean (s.e.m.), unless indicated otherwise. The number of biological (nontechnical) replicates and the specific statistical analyses used are detailed in the figure legends. Data distribution normality was tested using the Shapiro-Wilk normality test. Outliers were not excluded from the analysis. Comparisons between two unpaired groups were performed using the parametric Student’s *t*-test. For multiple comparisons, two-way ANOVA was used. P values are indicated as ∗: P ≤ 0.05, ∗∗: P ≤ 0.01, ∗∗∗: P ≤ 0.001, and ∗∗∗∗: P ≤ 0.0001 in all figures. For statistical analysis of scRNA-seq datasets, see method details.

## Supplemental information

Document S1. Figures S1–S9

Table S1. scRNA-seq analysis of MC38 tumors in D^KO^ and D^WT^ mice The data show the top 100 marker genes in the main cell populations; the differential gene expression between D^KO^ vs. D^WT^ tumors in the main cell populations; and the top 100 marker genes in the MonoMac subpopulations.
