# Trajectories of macrophage ontogeny and reprogramming in cancer

**DOI:** 10.1016/j.isci.2025.112975

**Published:** 2025-06-30

**Authors:** Florent Duval, Joao Lourenco, Mehdi Hicham, Gaël Boivin, Alan Guichard, Celine Wyser-Rmili, Nadine Fournier, Nahal Mansouri, Michele De Palma

## Main text

(iScience *28*, 112498, May 16, 2025)

In the original published version of this article, the heatmaps in Figures 4G and 8F were duplicates. This error was introduced at the proof correction stage after article acceptance, when higher-resolution heatmaps were supplied and the heatmap in Figure 4G was mistakenly supplied for Figure 8F as well. Thus, the corrected version of Figure 8 has been provided here and also updated in the published article. The discussion and conclusions of this paper remain unchanged after the correction. The authors apologize for any inconvenience caused to the readers.

**Figure:**
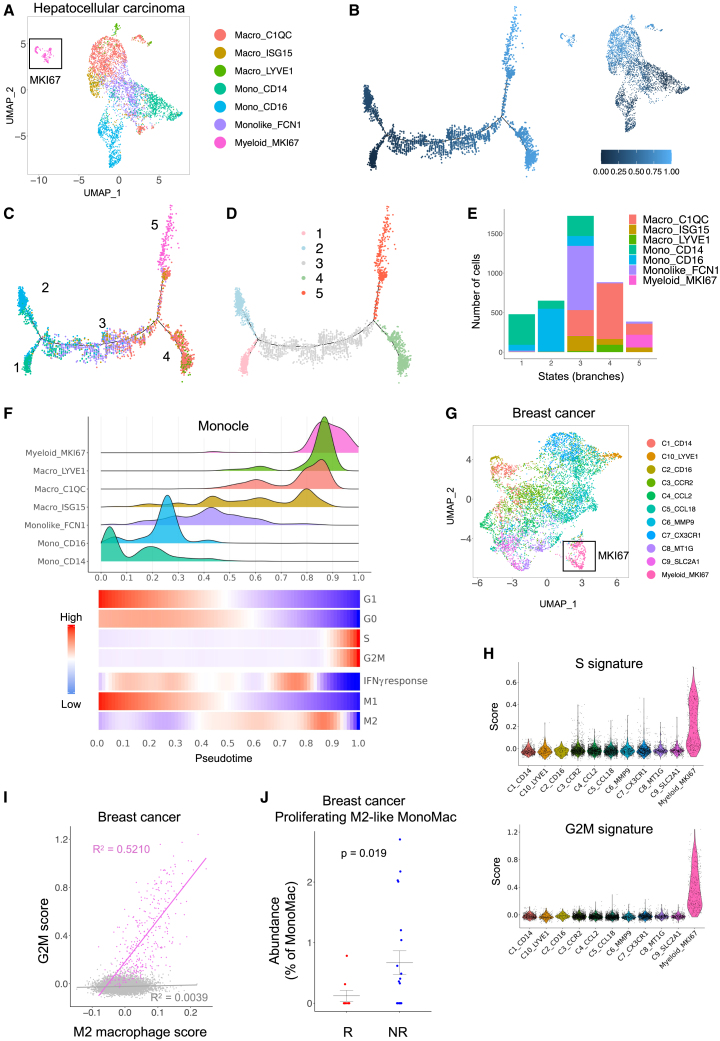
Figure 8. Cycling/M2-like macrophages are conserved in human cancer (corrected)

**Figure:**
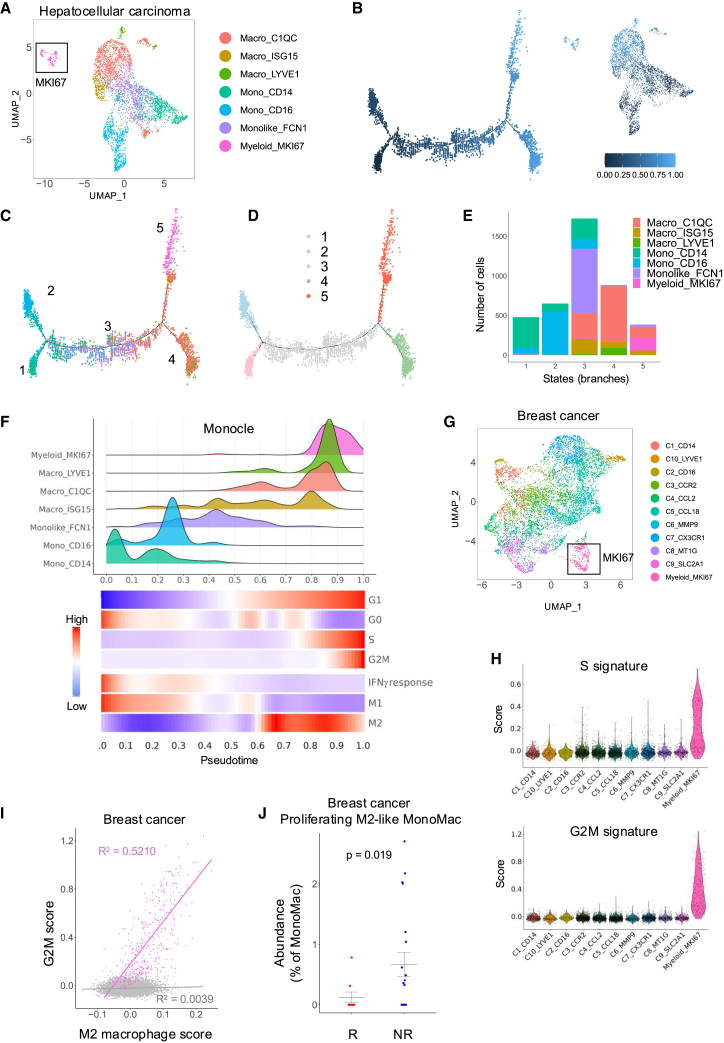
Figure 8. Cycling/M2-like macrophages are conserved in human cancer (original)

