# Supplementary material for: Trajectories of macrophage ontogeny and reprogramming in cancer
Source: iScience. 2025 Apr 22;28(5):112498. doi: 10.1016/j.isci.2025.112498 (PMC12139442; doi:10.1016/j.isci.2025.112498)
Supplement: Document S1. Figures S1–S9 [file mmc1.pdf]

iScience, Volume *28*

Supplemental information

Trajectories of macrophage ontogeny and reprogramming in cancer

Florent Duval, Joao Lourenco, Mehdi Hicham, Gaël Boivin, Alan Guichard, Celine Wyser-Rmili, Nadine Fournier, Nahal Mansouri, and Michele De Palma

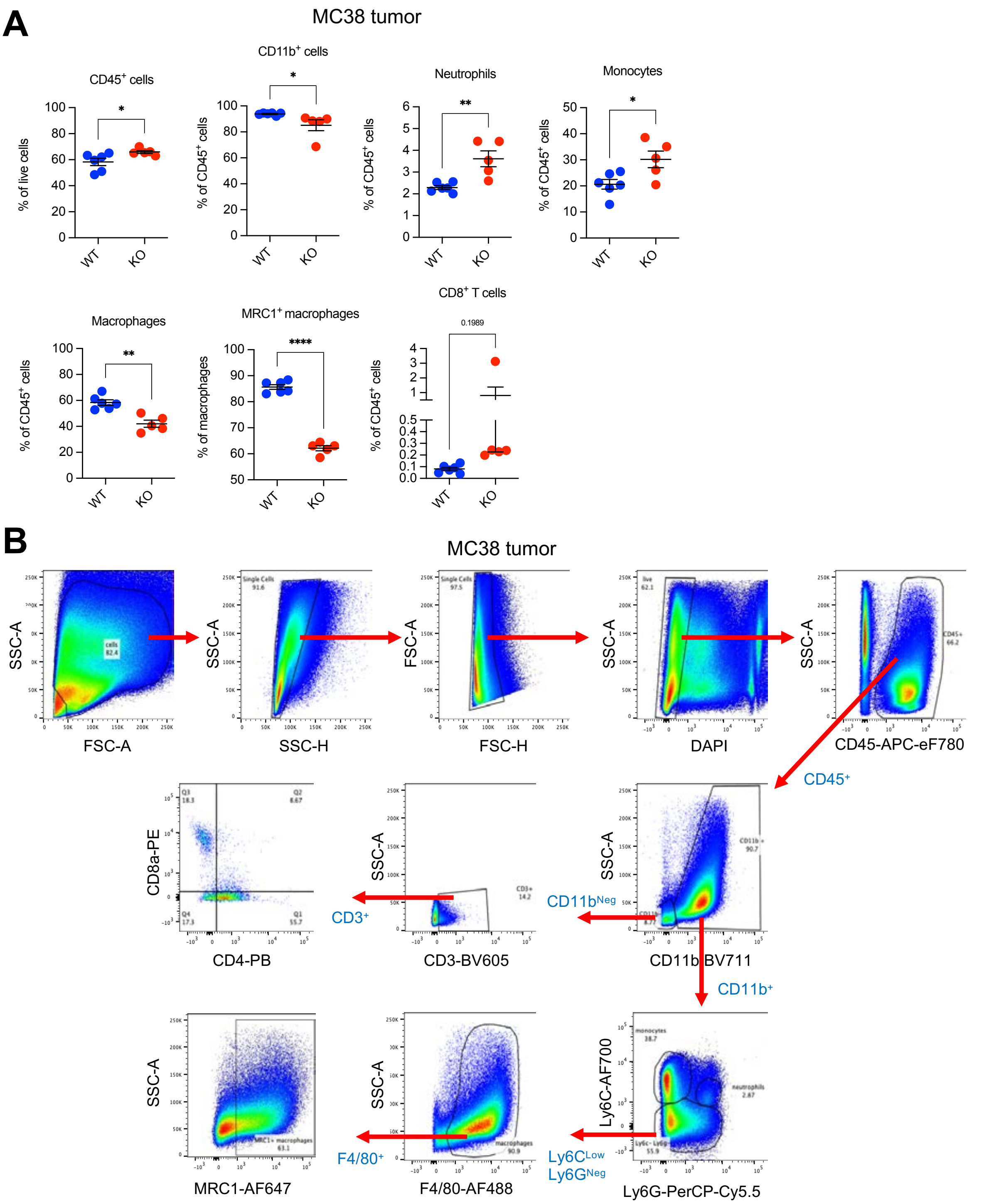

Fig S1. Myeloid-specific *Dicer1* inactivation reprograms the TME, Related to Fig. 1.

A. Percentage of the indicated cell types (mean ± s.e.m.) in MC38 tumors of D^{WT} (*n=6*) and D^{KO} (*n=5*) mice, measured by flow cytometry. Statistical analysis by unpaired Student's *t*-test.

B. Representative flow cytometry gating strategy used to identify immune cells in MC38 tumors.

*: P ≤ 0.05, **: P ≤ 0.01, and ****: P ≤ 0.0001.

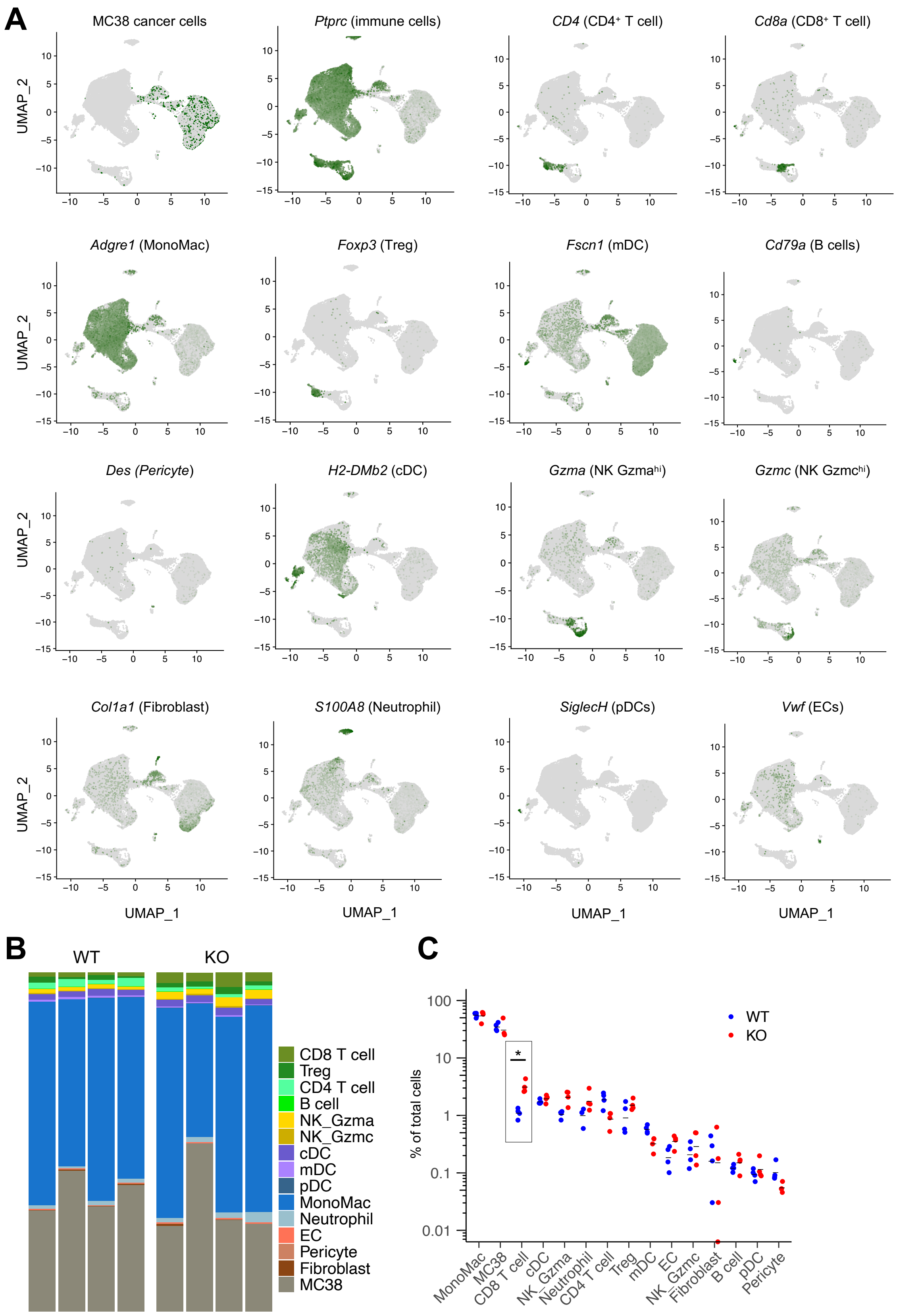

Fig S2. Cell composition of D^{WT} and D^{KO} tumors, Related to Fig. 2.

A. UMAP showing the expression of selected marker genes in MC38 tumors of D^{WT} and D^{KO} mice (pooled data; *n=4* per group) by scRNA-seq.

B. Proportions of each cell population in MC38 tumors of D^{WT} and D^{KO} mice (*n=4*).

C. Percentage of each cell population in MC38 tumors of D^{WT} and D^{KO} mice (*n=4*). Horizontal lines indicate the mean. $CD8^+$ T cells are boxed. Statistical analysis by unpaired Student's *t*-test.

*: $P \leq 0.05$.

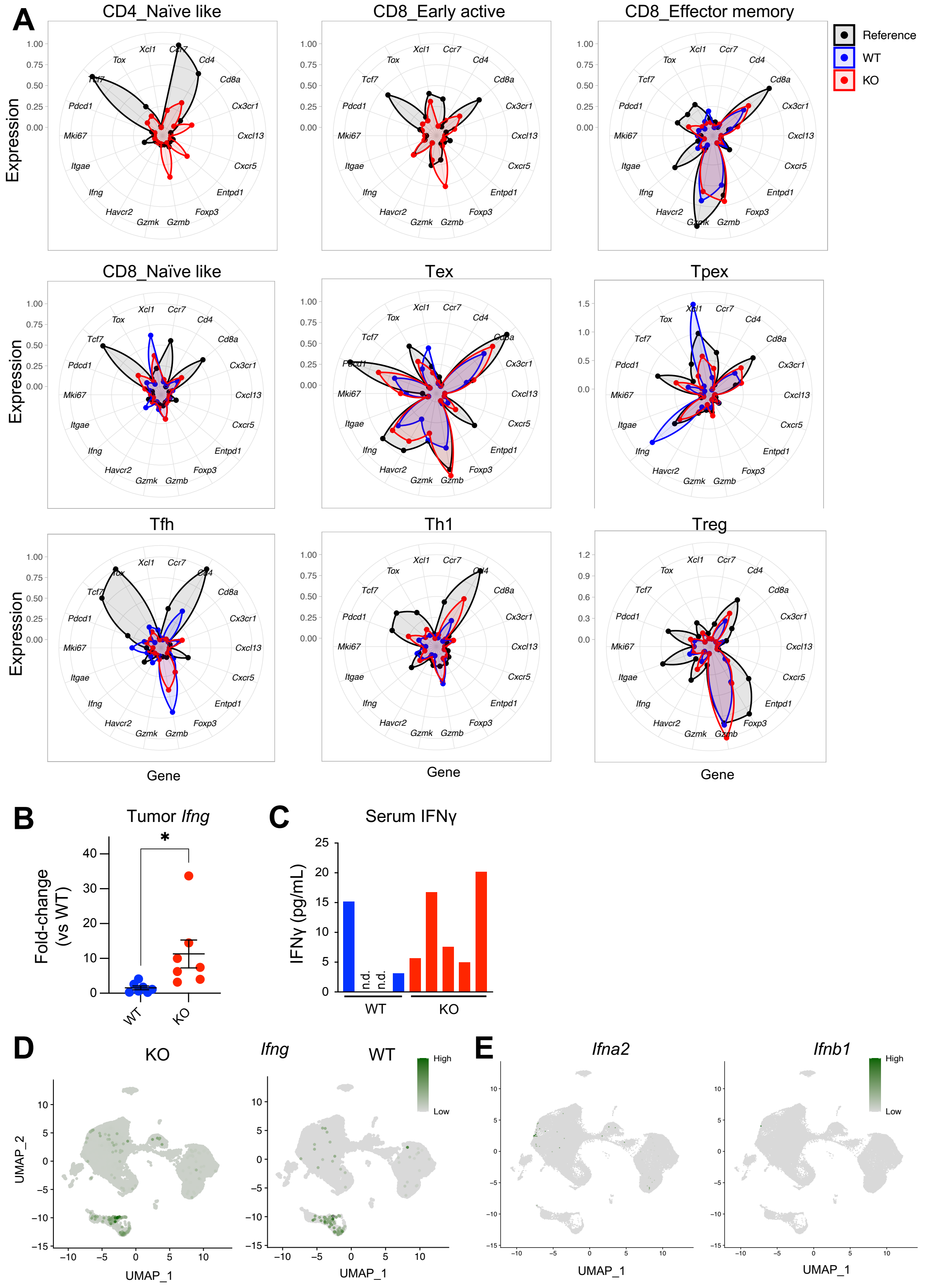

Fig S3. T cell subsets and IFNγ expression in D^{WT} and D^{KO} tumors, Related to Fig. 2.

A. Radar plots representing the normalized average expression of selected genes in each T cell subset from MC38 tumors of D^{WT} and D^{KO} mice (pooled data; *n=4* per group), according to the ProjecTILs murine reference map. Default threshold of at least 10 cells implies that that WT for CD4_Näive like and CD8_Early active is not displayed.

B. mRNA expression of *Ifng* in tumor lysates from D^{WT} and D^{KO} mice (*n*=*7*). Expression was normalized to the *Gapdh* housekeeping gene. Data are shown as fold-change in D^{KO} versus D^{WT} tumors. Statistical analysis by unpaired Student's *t*-test.
C. IFNγ protein concentration measured by ELISA in the serum of individual tumor-bearing D^{WT} and D^{KO} mice (*n=4-5*). N.d., not detectable.
D. UMAP showing expression of the *Ifng* gene in D^{WT} and D^{KO} tumors (*n*=*4*).
E. UMAP showing expression of the *Ifna2 and Ifnb1* genes in D^{WT} and D^{KO} tumors (pooled data, *n=4* per group).

*: P ≤ 0.05.

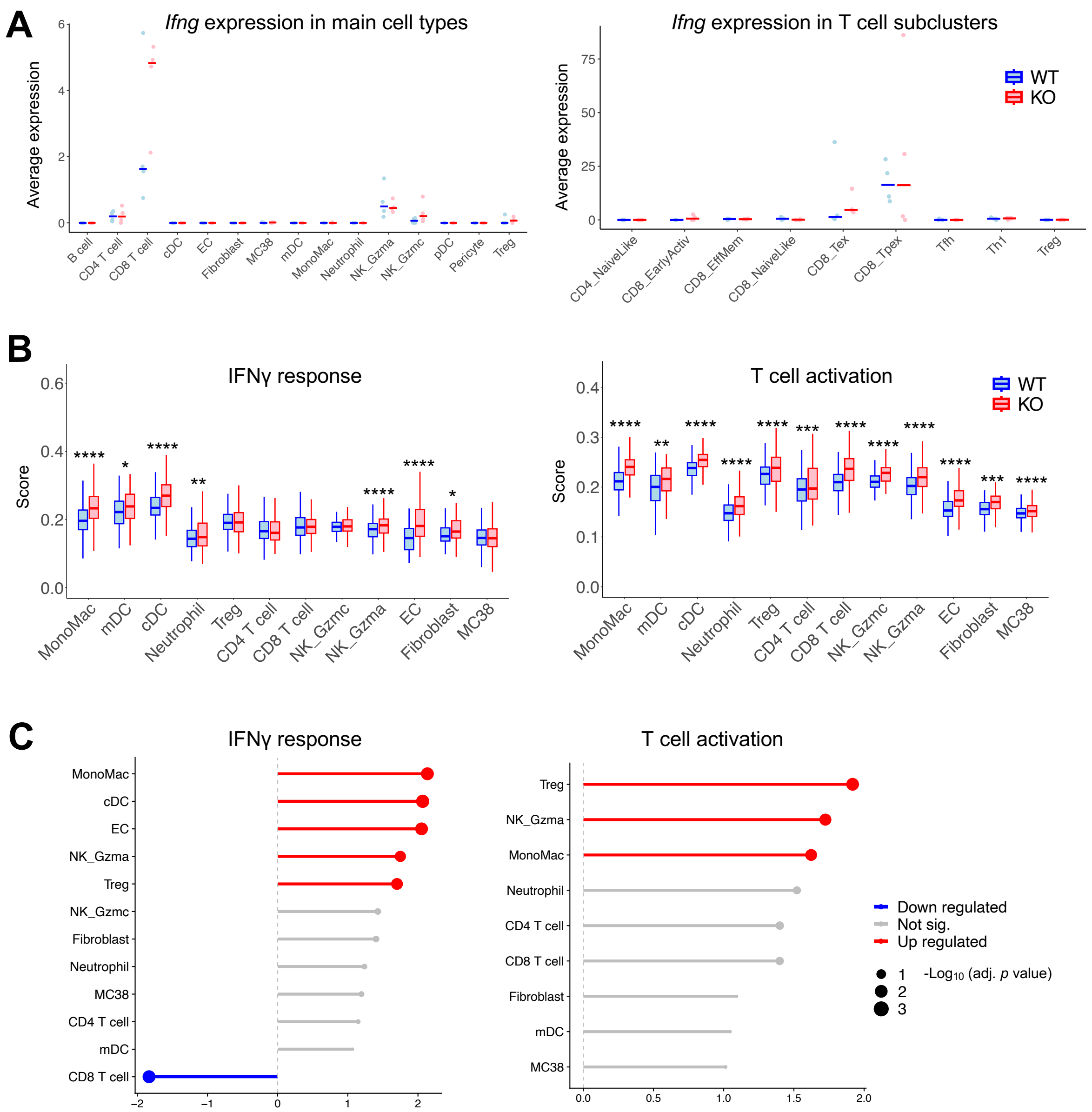

Fig S4. D^{KO} reprograms the TME through IFNγ, Related to Fig. 2.

A. Expression of the *Ifng* gene in the main cell types (left) and in each T cell subset (right) of MC38 tumors of D^{WT} and D^{KO} mice (*n=4* per group), Horizontal lines indicate the median.

B. Hallmark gene-set enrichment scores for the indicated biological processes in the main cell populations of tumors of D^{WT} and D^{KO} mice (*n=4*). Boxes represent the interquartile range (IQR), horizontal lines represent the median, and whiskers represent the range within 1.5 times the IQR. Statistical analysis by Student's *t*-test.

C. Lollipop plots showing Hallmark gene set enrichment analysis (GSEA) of the "IFNγ response" (left) and "T cell activation" pathways (right) across major cell populations. Genes were ranked by log_2 fold change between MC38 tumors of D^{KO} and D^{WT} mice

(*n=4*). P values were adjusted for multiple testing using the Benjamini-Hochberg method.

*: P ≤ 0.05, **: P ≤ 0.01, ***: P ≤ 0.001, and ****: P ≤ 0.0001.

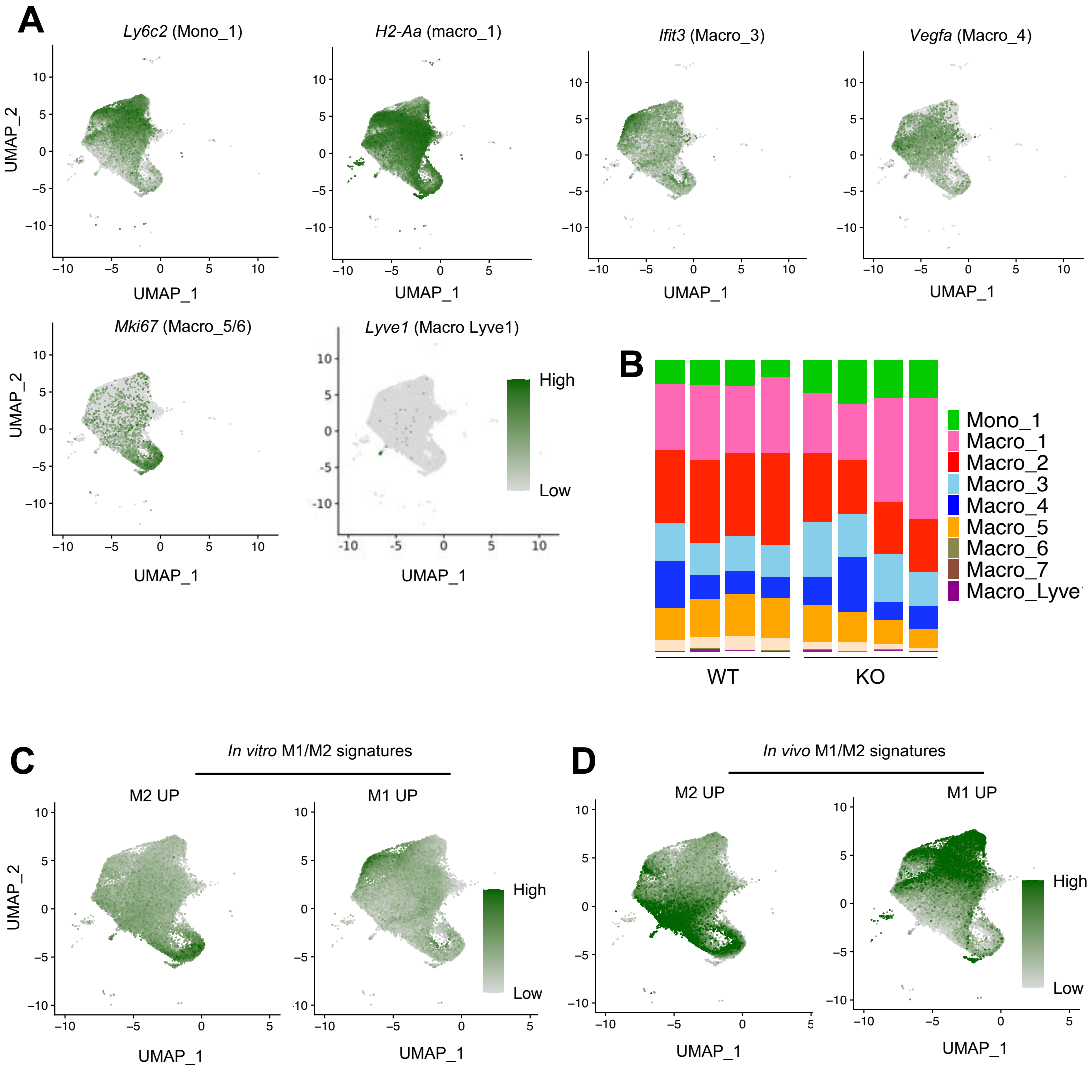

Fig S5. D^{KO} alters the abundance of inflammatory/M1-like and cycling/M2-like macrophages in tumors, Related to Fig. 3.

A. UMAP showing the expression of selected genes in the MonoMac clusters of MC38 tumors of D^{WT} and D^{KO} mice (pooled data; *n=4*).
B. Proportions of each MonoMac subpopulation in MC38 tumors of D^{WT} and D^{KO} mice (*n=4*).
C. UMAP showing M1/M2-like signature scores, derived from an *in vitro* dataset (see Fig. 3), in MonoMac subpopulations of MC38 tumors of D^{WT} and D^{KO} mice (pooled data; *n=4*).
D. UMAP showing M1/M2-like signature scores, derived from an *in vivo* dataset (see Fig. 3), in MonoMac subpopulations of MC38 tumors of D^{WT} and D^{KO} mice (pooled data; *n=4*).

*: P ≤ 0.05, **: P ≤ 0.01, ***: P ≤ 0.001, and ****: P ≤ 0.0001.

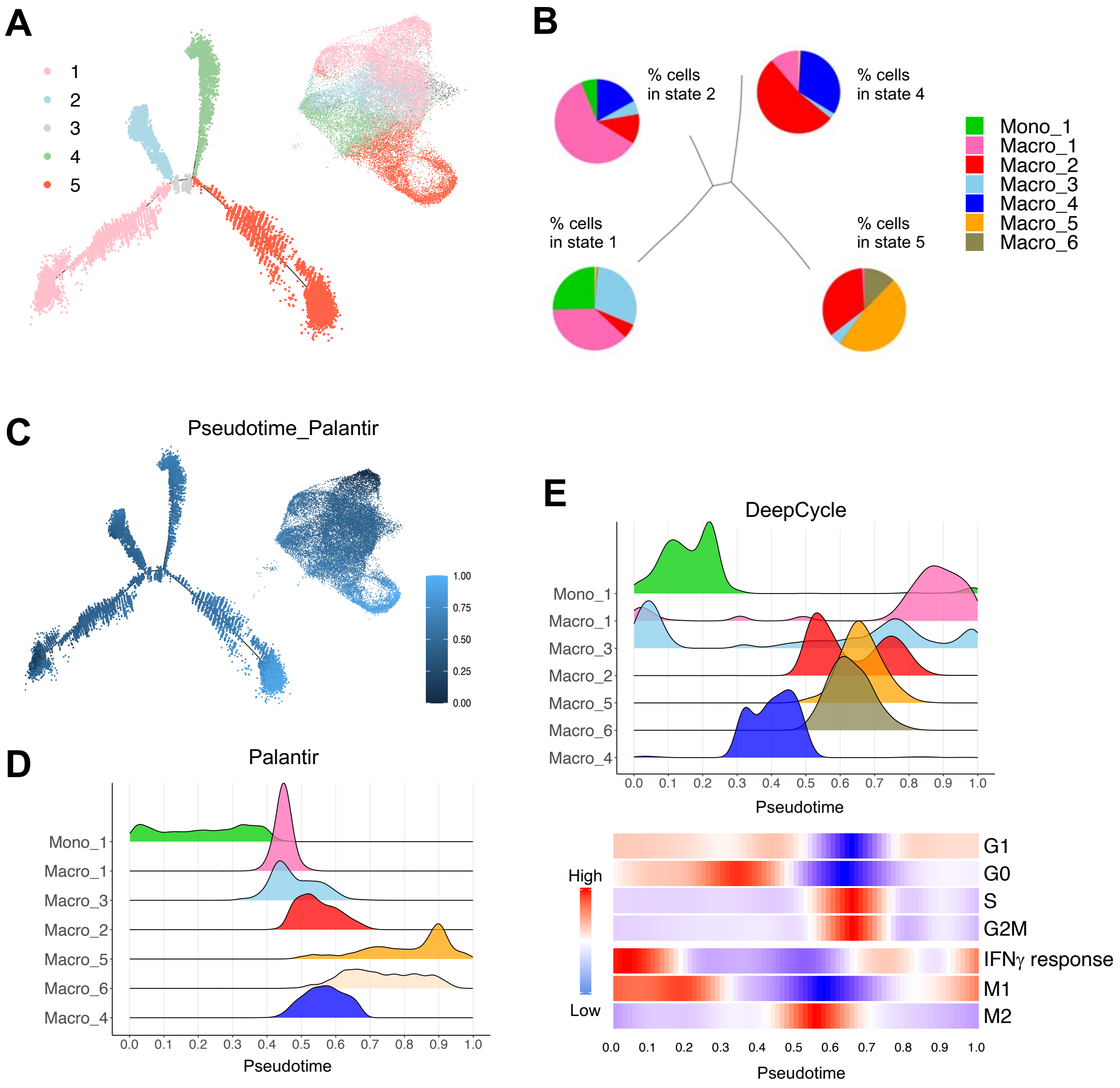

Fig S6. Dynamics analysis links trajectories and M1/M2 phenotypes in MonoMacs, Related to Fig. 4

A. Left: inferred trajectory states ("branches"; 1-5) of MonoMacs in MC38 tumors from D^{WT} and D^{KO} mice (pooled data; *n=4*). Right: projection of the identified trajectory states onto the UMAP.
B. Pie charts showing the relative proportions (percentage of cells) of MonoMac subpopulations across states ("branches") of the inferred Monocle trajectory in MC38 tumors of D^{WT} and D^{KO} mice (pooled data; *n=4*).
C. Monocyte-macrophage differentiation trajectory analysis by Palantir. Left: relative pseudotime along the differentiation trajectory determined by Monocle. Right: UMAP representation of the inferred pseudotime. The scale (0 to 1) corresponds to pseudotime.

D. Density distribution of MonoMac subpopulations along the differentiation trajectory inferred by Palantir.

E. Top: density of cells in each MonoMac subpopulation from MC38 tumors of D^{WT} and D^{KO} mice (pooled data; *n=4*), relative to transcriptional phase θ generated by DeepCycle. Bottom: heatmap showing the score of cell-cycle phases, M1/M2 macrophage phenotypes, and Hallmark IFNγ response gene set signatures, relative to transcriptional phase θ generated by DeepCycle. M1/M2-like macrophage phenotype signatures were derived from an *in vivo* dataset (see Fig. 4). Colors indicate signature scores, after conditional means smoothing and zero-centering.

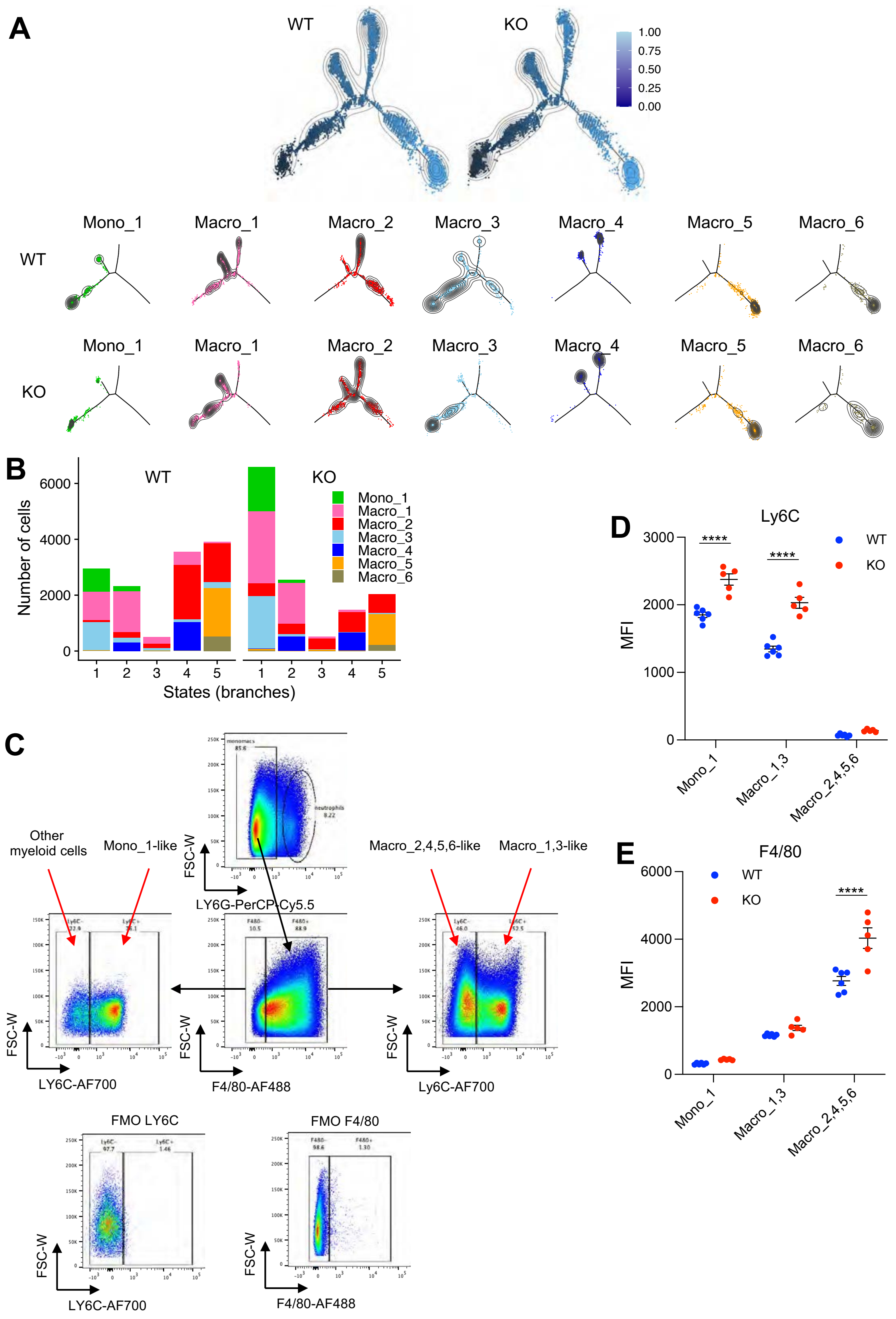

Fig. S7. D^{KO} interferes with MonoMac trajectories and stalls them in a cell cycle-arrested state, Related to Fig. 5.

A. Top: distribution of relative pseudotime along the monocyte-macrophage differentiation trajectory, inferred by Monocle. Concentric lines indicate the level of cell

density. The scale (0 to 1) corresponds to pseudotime. Bottom: density of cells from each MonoMac subpopulation across the monocyte-macrophage differentiation trajectory inferred by Monocle. Concentric lines indicate the level of cell density. Data are from MC38 tumors of D^{WT} and D^{KO} mice (*n=4*).

B. Barpot indicating the number of cells in the MonoMac subpopulations, resolved into each state of the monocyte-macrophage differentiation trajectory inferred by Monocle, in MC38 tumors of D^{WT} and D^{KO} mice (*n=4*).

C. Representative flow cytometry gating strategies used to identify Mono_1-like, Macro_1/3-like and Macro_2/4/5/6-like subsets in MC38 tumors. Note that CD45 and CD11b were used to identify the parental myeloid populations, but are not displayed.

D. Mean fluorescence intensity (MFI) of Ly6C (mean ± s.e.m.) in the indicated MonoMac subsets from MC38 tumors of D^{WT} (*n=6*) and D^{KO} (*n=5*) mice. Statistical analysis by two-way Anova with Sidak's multiple comparison test.

E. MFI of F4/80 (mean ± s.e.m.) in the indicated MonoMac subsets from MC38 tumors of D^{WT} (*n=6*) and D^{KO} (*n=5*) mice. Statistical analysis by two-way Anova with Sidak's multiple comparison test.

****: P ≤ 0.0001.

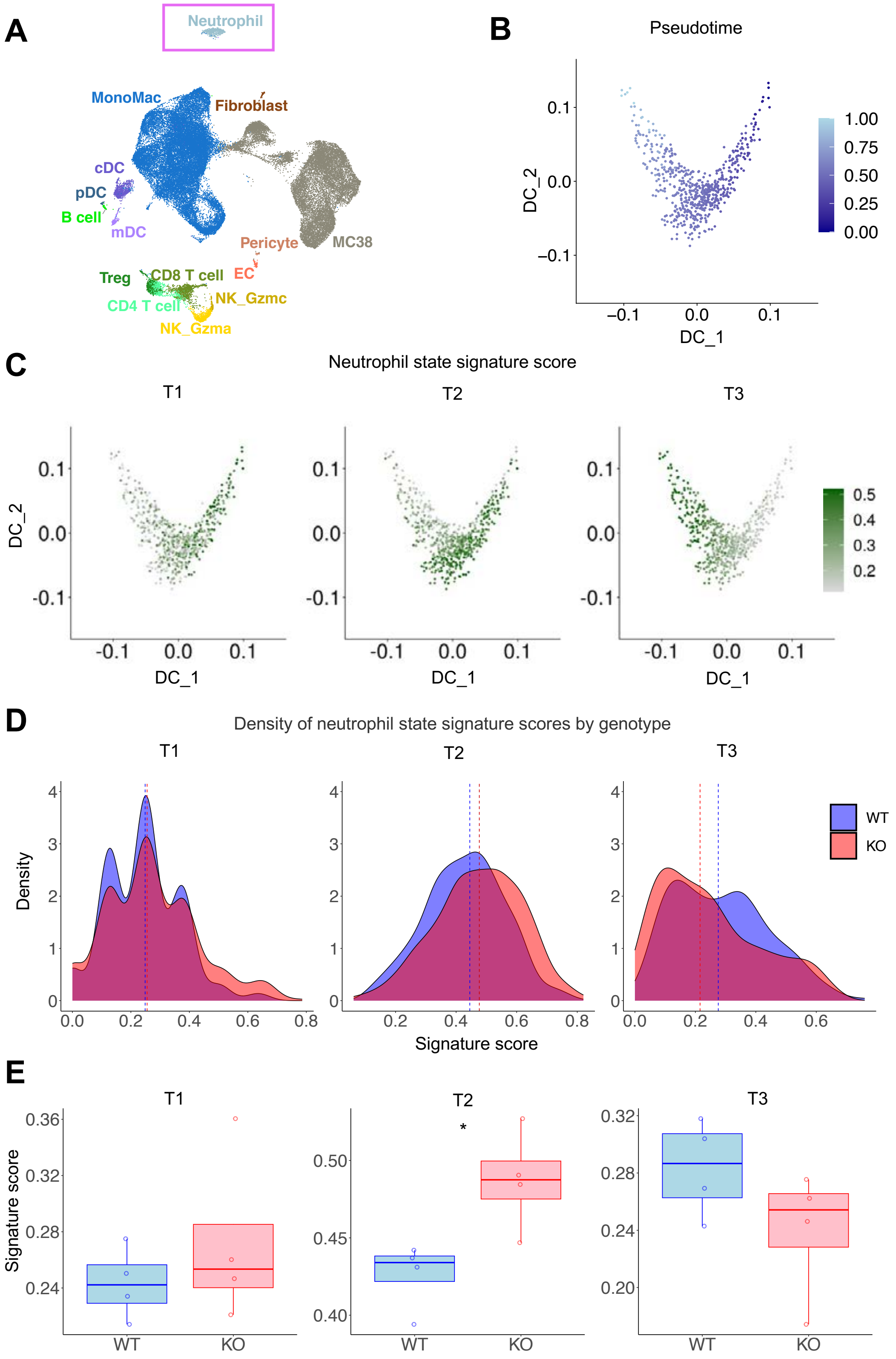

Fig S8. D^{KO} restrains protumoral neutrophil differentiation, Related to Fig. 5.

A. UMAP showing cell populations identified in MC38 tumors of D^{WT} and D^{KO} mice (pooled data; *n=4* per group) by scRNA-seq. Neutrophils are boxed.

B. Distribution of relative pseudotime along the neutrophil differentiation trajectory, inferred by Monocle, in MC38 tumors of D^{WT} and D^{KO} mice (*n=4*). The scale (0 to 1) corresponds to pseudotime.

C. Neutrophil state signature score enriched in each state (T1-3) of the neutrophil differentiation trajectory inferred by Monocle, in MC38 tumors of D^{WT} and D^{KO} mice (*n=4*). The scale (0 to 1) corresponds to pseudotime.

D. Density of each neutrophil state signature score in MC38 tumors of D^{WT} and D^{KO} mice (*n=4*).

E. Neutrophil state signature scores compared between D^{WT} and D^{KO} mice (*n=4*). Boxes represent the IQR, horizontal lines represent the median, and whiskers represent the range within 1.5 times the IQR. Statistical analysis by Student's *t*-test.

*: $P \leq 0.05$.

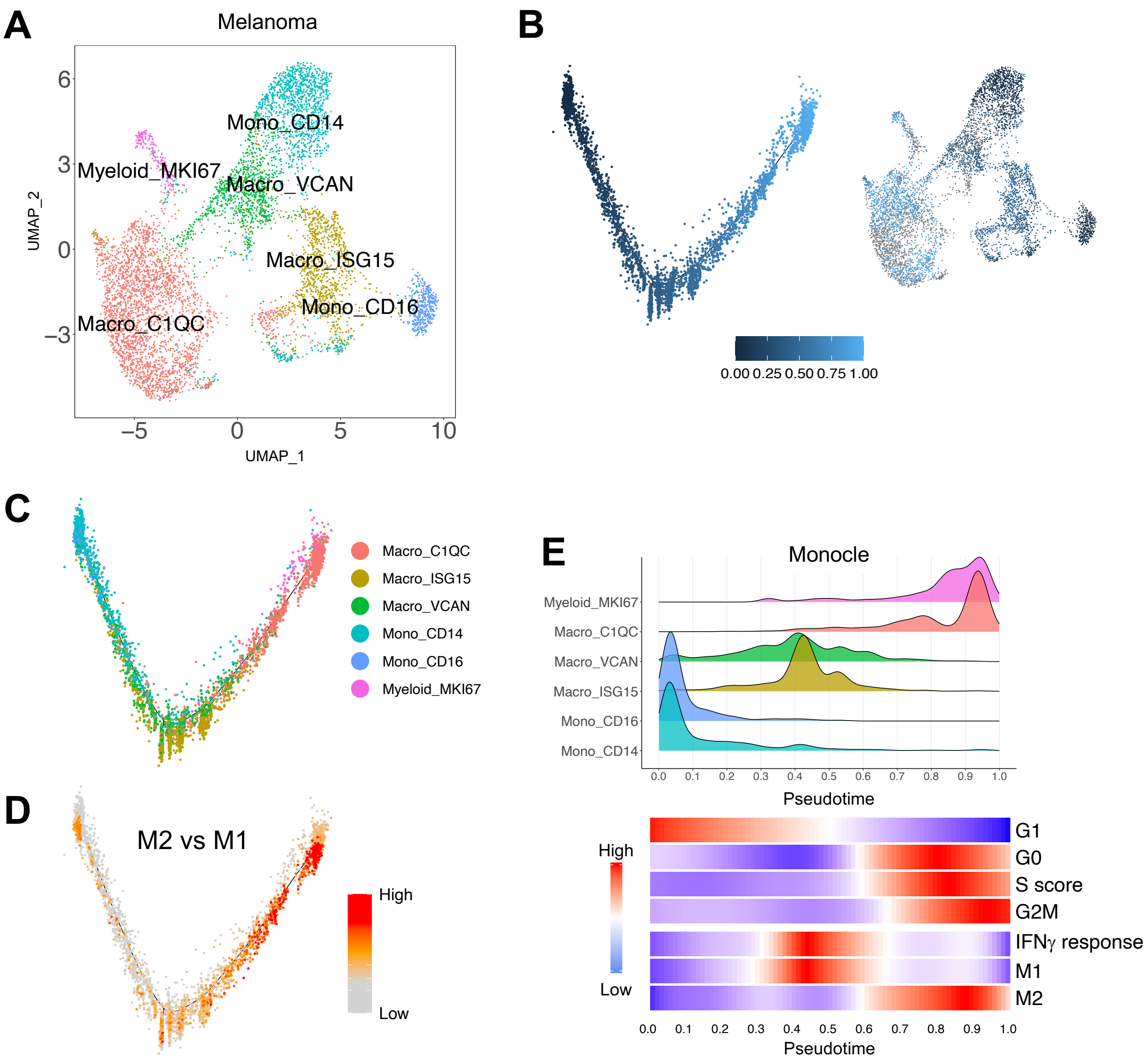

Fig S9. Cycling/M2-like macrophages are conserved in human cancer, Related to Fig. 8.

A. UMAP projection of MonoMacs in the human melanoma dataset. Cells are color coded by sub-population. Cycling macrophages are indicated as “Myeloid_MKI67”.
B. Left: relative pseudotime along the monocyte-macrophage differentiation trajectory, inferred by Monocle. Right: UMAP representing the relative pseudotime in the MonoMac population of the human melanoma dataset. The scale (0 to 1) corresponds to pseudotime.
C. Distribution of cells from each MonoMac subpopulation across the monocyte-macrophage differentiation trajectory, inferred by Monocle, in the human melanoma dataset. Colors indicate subpopulations of MonoMacs.
D. Scores for the signature of M2-like macrophage phenotype (derived from an *in vivo* dataset; see Fig. 3) plotted along the monocyte-macrophage differentiation trajectory inferred by Monocle.

E. Top: plot representing the density of cells in each MonoMac subpopulation from the human melanoma dataset relative to monocyte-macrophage differentiation pseudotime. Bottom: heatmap showing the score of cell-cycle phases, M1/M2-like macrophage phenotypes and Hallmark IFNγ response gene-set signatures relative to monocyte-macrophage differentiation pseudotime. M1/M2-like macrophage phenotype signatures were derived from an *in vivo* dataset (see Fig. 3). Colors indicate enrichment scores, after conditional means smoothing and zero-centering.
